# Susceptibility of amphibians to chytridiomycosis is associated with MHC class II conformation

**DOI:** 10.1098/rspb.2014.3127

**Published:** 2015-04-22

**Authors:** Arnaud Bataille, Scott D. Cashins, Laura Grogan, Lee F. Skerratt, David Hunter, Michael McFadden, Benjamin Scheele, Laura A. Brannelly, Amy Macris, Peter S. Harlow, Sara Bell, Lee Berger, Bruce Waldman

**Affiliations:** 1 Laboratory of Behavioral and Population Ecology, School of Biological Sciences, Seoul National University, Seoul 151–747, South Korea; 2 School of Public Health, Tropical Medicine and Rehabilitation Sciences, James Cook University, Townsville, Queensland 4811, Australia; 3 New South Wales Office of Environment and Heritage, Biodiversity Conservation Section, Queanbeyan, New South Wales 2620, Australia; 4 Taronga Conservation Society Australia, Herpetofauna Division, Mosman, New South Wales 2088, Australia; 5 Fenner School of Environment and Society, Australian National University, Acton, Australian Capital Territory 2601, Australia

**Keywords:** conservation, disease resistance, emerging infectious disease, major histocompatibility complex, peptide-binding domain, pocket residue

## Abstract

The pathogenic chytrid fungus *Batrachochytrium dendrobatidis* (Bd) can cause precipitous population declines in its amphibian hosts. Responses of individuals to infection vary greatly with the capacity of their immune system to respond to the pathogen. We used a combination of comparative and experimental approaches to identify major histocompatibility complex class II (MHC-II) alleles encoding molecules that foster the survival of Bd-infected amphibians. We found that Bd-resistant amphibians across four continents share common amino acids in three binding pockets of the MHC-II antigen-binding groove. Moreover, strong signals of selection acting on these specific sites were evident among all species co-existing with the pathogen. In the laboratory, we experimentally inoculated Australian tree frogs with Bd to test how each binding pocket conformation influences disease resistance. Only the conformation of MHC-II pocket 9 of surviving subjects matched those of Bd-resistant species. This MHC-II conformation thus may determine amphibian resistance to Bd, although other MHC-II binding pockets also may contribute to resistance. Rescuing amphibian biodiversity will depend on our understanding of amphibian immune defence mechanisms against Bd. The identification of adaptive genetic markers for Bd resistance represents an important step forward towards that goal.

## 1. Introduction

The emerging infectious skin disease chytridiomycosis, caused by the chytrid fungus *Batrachochytrium dendrobatidis* (denoted Bd) [1,2], is a primary driver of global amphibian population declines and species extinctions [3,4]. Bd colonizes the keratinized epithelial cells of its host's skin [2]. In susceptible amphibians, acute infections can disrupt membrane potentials, leading to paralysis and heart failure [5]. Bd has been detected in more than one-third of all amphibian species surveyed around the world [3,4]. Disease progression, and whether infection culminates in morbidity or mortality, depends on many factors, especially the capacity of the host immune system to respond to the infection [6]. Thus, even in the midst of epizootics, variation exists among individuals in their vulnerability to the disease. In amphibian communities, some species thrive even as others around them perish.

The major histocompatibility complex (MHC) encodes receptors at the cell surface that induce and regulate acquired immune responses against pathogens in all jawed vertebrates [7]. Resistance of amphibians to bacterial and viral diseases that cause population die-offs has been demonstrated to be conferred by particular MHC alleles [8,9]. A recent study identified possible MHC allele-specific disease resistance to chytridiomycosis [10]. These findings suggest that features of MHC molecules may improve their capacity to bind Bd antigens and induce adaptive immune responses. If the results have some generality, alleles that encode these molecules should be strongly selected in infected populations.

Many variable amino acid residue positions associated with disease susceptibility in vertebrates are situated in exon 2 of the MHC class II (MHC-II) B gene [11,12], which encodes the *β*1 segment of the antigen-binding groove that presents epitopes to T cells [7]. The stability of the antigen–MHC complex depends mainly on deep pockets within the binding groove that interact directly with antigen residues [12]. Amino acid changes in this segment result in important structural modifications of the binding groove that can change the affinity of the MHC molecule for particular antigens [11,12]. The MHC-II β1 domain of several amphibian species has been sequenced [10,13–16] and residue positions important for antigen binding may be under positive selection [10,15]. Analyses of Bd resistance among amphibians as a function of their MHC-II β1 domain offer a means to examine the evolution of resistance to Bd and to identify specific MHC conformations that confer Bd resistance.

Here, we use a combination of comparative and experimental approaches to demonstrate selection for MHC conformations that foster the survival of Bd-infected amphibians across four continents. We show that resistant amphibians share amino acids within the MHC-II binding groove. We assess how specific sites in this region affect the conformation and binding affinities of MHC molecules that are important in conferring Bd resistance. We then test our predictions by conducting laboratory challenge experiments on a threatened Australian tree frog species, comparing survivorship of MHC genotypes in populations that have evolved with Bd to those that remain naive to the pathogen.

## 2. Material and methods

### (a) Sample collection

In South Korea, we obtained 30 toe-clip samples from wild-caught Asiatic toads (*Bufo gargarizans*) in Geumsan (36°8.249′ N, 127°22.876′ E) and Jeonju (35°47.241′ N, 127°8.348′ E), and 10 samples from wild-caught oriental fire-bellied toads (*Bombina orientalis*) in Chuncheon (37°58.664′ N, 127°36.146′ E) and Chiaksan (37°23.676′ N, 128°03.221′ E) (electronic supplementary material, table S1). These two species historically have been infected by endemic Bd strains in Korea and show no evidence of morbidity nor mortality attributable to chytridiomycosis despite extensive fieldwork having been undertaken [17]. In Australia, we obtained toe-clip samples from 30 wild alpine tree frogs (*Litoria verreauxii alpina*) in each of three similar but geographically separate populations in Kosciuszko National Park, New South Wales. Two of the three populations were long-exposed to the pathogen (site A, Kiandra, 35°52.335′ S, 148°29.994′ E; site B, Ogilvies Creek, 36°2.175′ S, 148°19.327′ E) and a third never had been exposed to Bd (site C, Grey Mare Range, 36°19.010′ S, 148°15.567′ E).

### (b) MHC-II β1 genotyping and structure comparison

DNA was extracted from toe clips using a salting-out extraction method with ammonium acetate [18]. We used existing primers for *B. gargarizans* and newly designed primers (see detailed methods in electronic supplementary material) for *L. v. alpina* and *B. orientalis* to genotype samples at the β1 domain of one MHC-II locus. Full details of primer sequences and PCR protocols are given in electronic supplementary material, table S2. MHC-II β1 amplicons were purified and cloned using the RBC A&T cloning kit and accompanying HIT-DH5 α competent cells (RC001 and RH617, RBC Bioscience, Taipei, Taiwan) following the manufacturer's protocol. Between 8 and 20 clones were amplified by PCR (electronic supplementary material, table S2) and sequenced using M13 primers. Allelic identity was confirmed when the corresponding DNA sequence was found in at least two clones. Only one or two alleles were recovered from any of our samples, confirming that our primers targeted one single MHC-II locus in all species.

Sequences obtained were aligned with MHC-II β1 sequences of 17 amphibian species from around the world that vary in susceptibility to Bd [19–25] (length of sequences: 37–81 amino acids; complete list in electronic supplementary material, figure S1; GenBank accession numbers KJ679288–KJ679331) using Clustal W [26]. Sequences then were translated into amino acids using BioEdit [27]. We examined the amino acid composition at 15 codon positions known to affect the properties of the P4 (seven sites), P6 (four sites) and P9 (four sites) pockets of the MHC-II peptide-binding groove in humans [11,12]. Codon β9 of pocket P9 was not evaluated because it was missing from more than half of our dataset. The most frequent amino acid across MHC-II β1 sequences of Bd-resistant amphibians was recorded for each of the 15 positions.

We hypothesized that these most frequent amino acid compositions represent pocket conformations associated with Bd resistance. Allelic frequencies within populations could not be considered because such data are not available for most amphibian species. Nevertheless, if Bd induced strong selection for particular MHC-II conformations, we would expect to find a trend. Failure to find a relationship, however, would not allow us to draw any conclusion. For each MHC-II binding pocket, the proportion of β1 sequences containing all the amino acids most frequent in resistant amphibians was compared with that characteristic of susceptible species. Statistical significance was assessed by two-sample tests of equality of proportions, correcting for multiple tests with the Benjamini and Hochberg's procedure [28], using the statistical platform R v. 3.0.2 [29].

### (c) Microsatellite genotyping and data analysis

A panel of nine microsatellite markers, including two markers isolated from the *Litoria ewingii* complex [30] and seven from a *L. v. alpina* partial genomic DNA library (see the electronic supplementary material), was organized into two fluorescently labelled multiplexes, and multiplex PCR was performed with Qiagen multiplex PCR master mix following the method described in electronic supplementary material, table S3 [31]. Details of microsatellite primers, GenBank accession numbers and PCR protocols are given in electronic supplementary material, table S3. Allele sizes were assigned using an ABI3730 DNA analyser at NICEM (Seoul National University, South Korea) and Peak Scanner v. 1.0 (Applied Biosystems, Carlsbad, CA, USA). Conformity to Hardy–Weinberg equilibrium and linkage disequilibrium was determined with Genepop v. 4.0 [32]. Heterozygosity values and frequency of null alleles were estimated with CERVUS [33].

Frequency of null alleles was high (*F* > 0.10) for four markers (*Livea-*GT1, *Livea*-AG3, Le2 and Le4) in the three populations genotyped. Therefore, we statistically adjusted allele frequencies assuming a single new allele size by generating a new genotyping dataset corrected for null alleles in FreeNA [34]. Subsequent analyses were run both with the five-marker dataset and the full nine-marker dataset corrected for null alleles.

### (d) Experimental infection

Fifteen clutches of *L. v. alpina* were collected from the three populations described above. Frogs were reared in Bd-free quarantine conditions to adulthood. Frogs were confirmed to be Bd-negative by qPCR [35] prior to the commencement of the exposure experiment. Two hundred adults were randomly chosen from each clutch and population to be assigned to treatments that were inoculated with 750 000 infective Bd zoospores (strain AbercrombieNP-L.booroolongensis-09-LB-P7). The experimental design, involving the utilization of frogs from each population and clutch together with details of the blind randomized block design used for allocation of treatment groups, is outlined in electronic supplementary material, table S4. As a control, another 56 frogs were sham-infected with culture medium in dilute salt solution. Numbers of frogs available for treatments were subject to actual clutch sizes and natural attrition during growth and development.

Frogs were maintained in a quarantine room at temperatures between 18 and 20°C under a 12 L : 12 D regimen. Subjects were housed individually in small plastic tubs on an angled rack with drainage holes, a loose pebble floor and a gauze-covered top. They were fed twice weekly with vitamin-dusted (calcium and herptivite alternately) crickets, and tubs were cleaned daily by flushing with fresh filtered water.

Subjects were monitored daily for clinical signs of infection (dullness, lethargy, peripheral erythema and increased skin shedding). Bd infection intensity data were collected weekly by swabbing all subjects, each with fresh gloves and a new sterile dry swab (MW100; Medical Wire and Equipment, Corsham, UK). Infection intensity was estimated as Bd zoospore equivalents (ZSE) in each swab sample using a TaqMan real-time qPCR assay [35] and standards with known Bd quantity. Individual swabs were analysed in triplicate with an internal positive control. Once the last surviving subjects effectively cleared themselves of Bd infection (0–10 ZSE) over four successive weeks, the experiment was terminated.

Frogs showing marked clinical signs of chytridiomycosis were euthanized throughout the experiment using tricaine methanesulfonate (MS-222). Subjects to be sacrificed first were swabbed to quantify Bd infection intensity by qPCR. Foot or toe-clip samples for MHC analysis were collected post-mortem soon after sacrificing subjects and were placed into 90% ethanol. We genotyped the MHC-II β1 domain in 100 *L. v. alpina* that we had infected with Bd. This selection included the only six individuals that survived the experiment and 94 individuals randomly chosen and encompassing all the clutches collected from the three populations (8–10 individuals per clutch; electronic supplementary material, table S4).

### (e) Survival statistical analysis

We used Cox proportional hazard models [36] to identify variables that affected survival of *L. v. alpina* during the experiment. Parameters included in the initial model were site of origin, clutch, maximum Bd infection load, mass at the start of the experiment, change in mass over the course of the experiment, MHC heterozygosity, allelic divergence (measured as *p*-distance) and pocket residue composition (table 1). We also examined two-way interaction terms between maximum infection load and the other variables, and between masses and the other variables sequentially. We used likelihood ratio tests to calculate the predictive power of each variable. Independent variables that did not show a significant association with length of survival were excluded from the model. The significance of each variable's effect on hazard risk was evaluated with Wald *z* statistics. Analyses were conducted with the ‘survival’ package in R v. 3.0.2.

**Table 1.** Amino acids affecting MHC class II peptide-binding pockets most common in amphibians resistant to infection by *Batrachochytrium dendrobatidis* (Bd). Proportions of MHC-II alleles with the targeted amino acid composition were calculated for worldwide Bd-resistant amphibians (Res. sp.), Bd-susceptible amphibians (Susc. sp.), *L. v. alpina* individuals that survived (Res. *Livea*) or died (Susc. *Livea*) from experimental Bd infection. The significance of the differences in proportion was calculated using two-sample tests for equality of proportions. Tyr/Phe, Ile/Leu, Arg/Lys, Asp/Glu: some sites could be any of two amino acids with the same chemical properties. Asterisk denotes *p*-values corrected for multiple comparisons using the Benjamini and Hochberg's procedure (see text).

MHC class II β1 pocket residues	Res. sp.	Susc. sp.	*p*	Res. *Livea*	Susc. *Livea*	*p*
Pocket 4 (Ser13β- Tyr/Phe26β- Ile/Leu67β- Gln70*β* -Arg/Lys71β- Ala74β- Tyr/Phe78β)	37/155 (0.239)	6/66 (0.909)	0.009 *(0.014)	0/8 (0.000)	0/14 (0.000)	n.a.
Pocket 6 (Val11β- Asp/Glu28β- Tyr/Phe30β- Asp/Glu66β)	42/155 (0.271)	0/72 (0.000)	<0.001 *(<0.001)	2/8 (0.250)	2/14 (0.143)	0.265 *(0.398)
Pocket 9 (Tyr/Phe37β- Pro56*β*- Asp/Glu57β- Tyr60β)	66/155 (0.426)	22/72 (0.306)	0.041 *(0.042)	8/8 (1.000)	6/14 (0.429)	0.004 *(0.012)

### (f) Detection of selection pressure

Genetic variation among populations (*F*_ST_) at MHC and microsatellite markers was calculated and compared with simulated expected *F*_ST_ values in wild *L. v. alpina* populations using Fdist [37] implemented in Lositan [38]. Markers with *F*_ST_ values significantly higher than the simulated distribution (posterior probability more than 0.95) were considered to be under positive selection. Analyses were done on the full dataset including the three populations, and on pairs of populations, to identify population-specific differences in selection pressure. Balancing or directional selection affecting MHC-II in wild *L. v. alpina* populations was also assessed using two tests based on deviation of allele frequencies from neutral expectation (Watterson's homozygosity test [39] and Slatkin's exact test [40]) conducted in Arlequin v. 3.5 [41].

Selection pressures on the MHC-II β1 domain were assessed by estimating the ratio of non-synonymous to synonymous nucleotide substitutions using multiple methods available on the Datamonkey website [42]. First, we confirmed the absence of recombination in our sequences using single breakpoint recombination (SBR) and genetic algorithms for recombination detection (GARD) [43]. To estimate the ratio of non-synonymous to synonymous nucleotide substitutions, we used the partitioning approach for robust inference of selection (PARRIS) [44] (*ω* = d*N*/d*S*; positive selection: *ω* > 1). Analyses were conducted across MHC-II β1 sequence alignments to identify the selection model best fitting our data. Positively selected sites were detected using single-likelihood ancestor counting (SLAC), fixed effects likelihood (FEL), random-effects likelihood (REL) analysis [45] and mixed-effects model of evolution (MEME) [46]. We also tested whether specific biochemical properties were driving substitutions at the peptide-binding sites using the property-informed model of evolution (PRIME) method [42], which includes the Holm–Bonferroni procedure to control for familywise false positives within sites.

## 3. Results

### (a) Comparison of MHC-II β1 in worldwide amphibians

**Figure 1.**
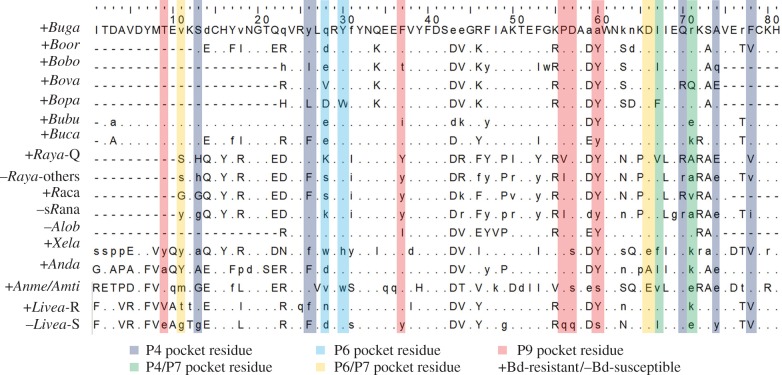
Alignment of the β1 domain of the MHC class II in amphibians illustrating peptide-binding residues. Each sequence in the alignment represents a consensus sequence grouping alleles isolated in a species, or in a subgroup within species. Susceptibility of each was determined by experimental infections or field observation of Bd-associated population declines. Positions encoding amino acids similar to Bd-resistant *Bufo gargarizans* are represented by a dot. Variable positions in consensus sequences are represented by the most frequent amino acid in the group (lower case letters). Dashes indicate missing data. Peptide-binding residues are highlighted in colours, as denoted, to indicate their association with pockets of the MHC peptide-binding groove. Bd-resistant *Litoria verreauxii alpina* (*Livea-*R), Bd-susceptible *L. v. alpina* (*Livea-*S), *Bufo gargarizans* (*Buga*), *Bombina orientalis* (*Boor*), *Bombina bombina* (*Bobo*), *Bombina variegata* (*Bova*), *Bombina pachypus* (*Bopa*), *Bufo bufo* (*Bubu*), *Bufo* [*Epidalea*] *calamita* (*Buca*), *Rana [Lithobates] yavapaiensis* (*Raya*), *Rana* [*Lithobates*] *catesbeiana* (*Raca*), susceptible *Rana* spp. (s*Rana*) including *Rana* [*Lithobates*] *clamitans*, *R. pipiens*, *R.* [*Lithobates*] *sylvatica*, *R.* [*Lithobates*] *warszewitschii* and *Rana temporaria*, *Alytes obstetricans* (*Alob*), *Xenopus laevis* (*Xela*), *Andrias davidianus* (*Anda*), *Ambystoma mexicanum* and *A. tigrinum* (*Anme/Amti*)*. Raya-*Q, allele from *Rana* [*Lithobates*] *yavapaiensis* associated with *Bd* resistance. The complete alignment with all MHC-II β1 included in the study is available in the electronic supplementary material.

The MHC-II β1 alleles of amphibian species least affected by Bd infection [21–25] consistently presented the same amino acids, or amino acids with similar chemical properties, at all 15 pocket residues (figure 1 and table 1; electronic supplementary material, figure S1). By contrast, these pocket-specific amino acid compositions were less frequent in species susceptible to Bd [19,20] (P4: 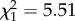, *p* = 0.009; P6: 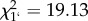, *p* < 0.001; P9: 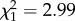, *p* = 0.04; table 1).

In wild populations of both *B. gargarizans* and *B. orientalis*, MHC-II alleles with the specific pocket 9 composition were more frequent than alleles with other P9 compositions (frequencies: *B. gargarizans* in Geumsan, 58.3%, 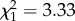, *p* = 0.03, in Jeonju, 81.6%, 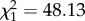; *p* < 0.0001; *B. orientalis* in Chuncheon, 70.0%, 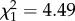, *p* = 0.02, in Chiak, 85.0%, 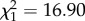, *p* < 0.0001; electronic supplementary material, table S1). By contrast, MHC-II alleles with the identified pocket 4 and pocket 6 compositions were equally or less frequent than other alleles for both species (frequencies: *B. gargarizans*, P4: 41.7%, *p* = 0.50, P6: 18.3%, *p* = 0.50; *B. orientalis*, P4 absent in all populations, P6: 37.5%*, p* = 0.48; electronic supplementary material, table S1).

### (b) Selection pressure and structural properties in MHC-II β1

We detected site-specific positive selection acting on MHC-II β1, including 14 of the 15 positions affecting the three peptide-binding pockets, across all amphibian groups (electronic supplementary material, table S5). We identified codon positions 37β and 56β of pocket P9 in *Bombina* and *Bufo* sp., and 26β of pocket 4 in *Bombina* sp., to be under purifying selection (electronic supplementary material, table S5). Variation at positions 13β and 57β was associated with a change in the chemical composition (property α1 in Conant–Stadler set of amino acid properties [47]) at these sites (13β: weight property α1 = −2.312, *p* = 0.01; 57β: α1 = −3.496, *p* = 0.05).

Electrically charged (e.g. negative Asp/Glu at 28β or 66β; positive Arg/Lys at 71β) or hydrophobic (e.g. Tyr/Phe at 26β, 30β or 37β) amino acids were found for 12 of the 15 codon positions for amphibians with low susceptibility to Bd (table 1). Amino acids with these properties can modulate anchor residue specificity by changing the charge characteristics of the pockets [12]. Other common amino acids, Valβ11 and Serβ13, increase the space available within the pockets for large anchor residues owing to their very small side chain [12]. Codon β56 encodes proline, the only such amino acid within the peptide-binding region of this MHC-II β locus (table 1; electronic supplementary material, figure S1). Proline is an amino acid with a unique conformational rigidity strongly affecting protein secondary structure such as alpha helices [48].

### (c) Experimental infection of *Litoria verreauxii alpina*

**Figure 2.**
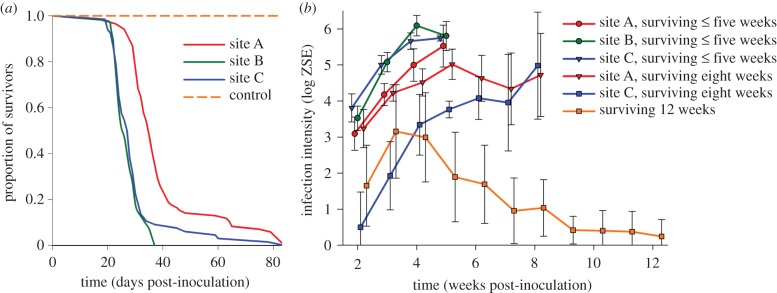
Experimental infection of *L. v. alpina* frogs from populations with varied Bd infection histories. (*a*) Survival curves for captive-reared frogs from two historically infected sites (A and B) and an uninfected site (C) in Kosciuszko National Park, Australia, experimentally infected with a virulent Bd culture. (*b*) Mean infection intensity as a function of time since Bd inoculation for those subjects that survived five weeks or less, eight weeks, or through the duration of the 12-week experiment. These results include the weekly swabs from up to 30 frogs from each site, selected by stratified random sampling based on days survived. Infection intensity is given in log-transformed ZSE. Error bars represent 95% confidence intervals.

After inoculating frogs with Bd, subjects from long-exposed site A survived significantly longer than those from the other long-exposed site B and naive site C (Kaplan–Meier *p* = 0.001; figure 2; electronic supplementary material, table S6). Many subjects were heavily infected and died within five weeks (figure 2). Infection loads of some individuals from sites A and C stabilized after five weeks but increased afterwards, leading to morbidity and mortality after eight weeks. Six of 200 frogs (five from site A, one from site C) demonstrated greatly reduced loads after three weeks and survived until the end of the experiment (figure 2). All non-exposed, control frogs survived until the end of the experiment.

**Figure 3.**
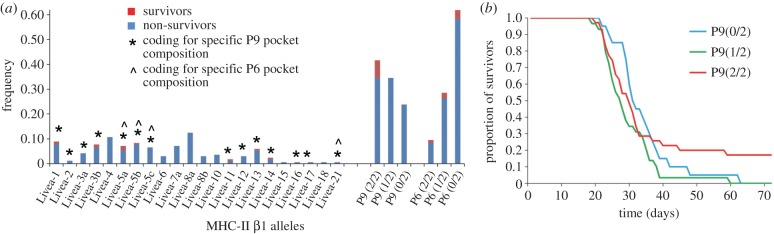
Association of the MHC class II β1 domain with survival to Bd in *L. v. alpina.* (*a*) Frequency of MHC class II β1 alleles (*Livea*) in subjects that survived (red) and succumbed (blue) to experimental infection by Bd, indicating alleles encoding the P9 and P6 pocket compositions most frequent in Bd-resistant amphibians; frequency of individuals with two (2/2), one (1/2) or no (0/2) MHC-II β1 alleles with the specific P9 residue composition. (*b*) Survival curves for individuals during the course of the Bd infection experiment with respect to the presence of the specific P9 pocket residue composition in two (2/2), one (1/2) or none (0/2) of their two MHC-II β1 domains.

We genotyped the β1 domain of one MHC-II locus in 84 individuals, including the six survivors, that we had inoculated with Bd. Twenty-two MHC-II β1 alleles were recovered, with eight alleles identified among the surviving individuals (*Livea-*1, 2, 3b, 5a, 5b, 11, 13 and 14; figure 3; electronic supplementary material, table S6 and figure S1). These eight alleles had identical residues at five codon positions associated with the P9 pocket, corresponding to the composition identified in resistant amphibians worldwide (table 1 and figure 1; electronic supplementary material, S1). Subjects that died had significantly lower frequencies of alleles with the specific P9 pocket composition than those that survived (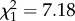, *p* = 0.004; table 1 and figure 3). None of the eight alleles had the P4 pocket composition identified in the worldwide MHC-II β1 alignment, and only two alleles had the targeted P6 pocket composition (table 1). We identified another 15 alleles in individuals that did not survive infection. Six of these alleles presented the P9 pocket composition identified in Bd-resistant amphibians (figures 1 and 3; electronic supplementary material, S1).

We used Cox proportional hazard models to determine whether P6 and P9 pocket residue composition, MHC heterozygosity and other variables affected the survivorship of subjects during the experiment. The model that best fitted the data included P6 and P9 pocket residue compositions, the clutch and site of origin, and the interaction between P9 composition and the maximum Bd infection load (LRT = 61.88, d.f. = 13, *p* < 0.0001; detailed results of the model in electronic supplementary material, table S7). Having the specific P9 pocket composition associated with Bd resistance in both MHC-II alleles significantly increased survival (*z* = −3.407, *p* < 0.001, figure 3; electronic supplementary material, table S7). Presence of two alleles with the targeted P6 composition had a significantly negative effect on survival (*z* = 3.070, *p* = 0.002). Having only one allele with the P6 or P9 compositions did not significantly improve survival (*z* = 0.864, *p* = 0.39, and *z* = −0.225, *p* = 0.82, respectively; electronic supplementary material, table S7). We did not observe any significant effect of MHC heterozygosity or allelic divergence on survival (LRT for model with MHC heterozygosity = 61.98, 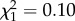, *p* = 0.75; LRT for model with allelic divergence = 65.27, 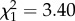, *p* = 0.07).

### (d) Selection pressure on *Litoria verreauxii alpina*

The best-fitting selection model allowed for site-specific positive selection (*ω* = 3.14, LRT = 6.58, *p* = 0.04). Along with other peptide-binding residues, residues β37 and β57 of the P9 pocket were identified as being under positive selection using REL analysis (posterior probability more than 0.99 for both residues). Residue β57 also was identified as being under positive selection using MEME (*p* = 0.002; electronic supplementary material, table S5).

If MHC alleles with the specific P9 pocket conformation are advantageous to *L. v. alpina* against Bd infection, we should also observe signs of directional selection pressure on this locus in wild *L. v. alpina* exposed to this pathogen. We sampled 30 individuals from each of the three sites used as source populations for the infection experiment. We genotyped these individuals for the MHC-II β1 domain and nine microsatellite markers. Contrary to our predictions, the frequency of P9-specific alleles in exposed site A (37.5%) was lower than that of other alleles (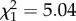, *p* = 0.50), whereas P9-specific alleles were more frequent in exposed site B (93.8%, 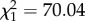, *p* < 0.0001) and in naive site C (97.5%, 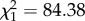, *p* < 0.0001). Levels of MHC heterozygosity were high in all populations (60–65%). Genetic variation among populations (measured as *F*_ST_) was significantly higher at the MHC-II locus than at the microsatellite loci among the populations (*F*_ST_ = 0.147, posterior probability more than 0.99; electronic supplementary material, table S7), suggesting that directional selection is affecting the MHC class II locus across populations [49]. However, Watterson's *F*-test and Slatkin's exact *p*-test did not detect any significant deviation from neutrality in MHC allele frequencies of the three populations (Watterson: *p* = 0.927 (site A), 0.337 (B), 0.336 (C); Slatkin: *p* = 0.912 (A), 0.162 (B), 0.556 (C)).

## 4. Discussion

We have shown that chytridiomycosis appears to select for specific properties of the MHC class II molecule P9-binding pocket. This is apparent both in our experimental infection study of *L. v. alpina* and our surveys of wild populations of Bd-resistant *Bufo* and *Bombina* species in Korea. While our comparative study also points to specific P4 and P6 pocket conformations that correlate with Bd resistance worldwide, these conformations did not improve survival of infected *L. v. alpina*, nor are they abundant in Korean *Bufo* and *Bombina* populations. Possibly, the importance of the various antigen-binding pockets in triggering an efficient immune response varies with hosts, infecting Bd strains and environmental factors.

The protective P9 pocket conformation includes an aromatic β37, an acidic Aspβ57, a Proβ56 and a hydrophobic β60 residue. We could not fully assess the importance of β9, another P9 peptide-binding residue, because the position is missing from sequences of many of the species that we examined. Residue Proβ56, although not directly binding to anchor residues of epitopes, may be of especial importance for efficient Bd antigen binding by influencing how the peptide-binding residue β57 is positioned within the P9 pocket [48].

Our experimental study on *L. v. alpina* demonstrates adaptive immune-system-mediated recovery during infection solely in individuals bearing two MHC alleles with the advantageous P9 conformation. MHC alleles are co-dominantly expressed [50], so two doses of MHC molecules binding preferentially to Bd peptides may be required to offset the pathogen's mechanism of attack on the adaptive immune system [51]. Alternatively, MHC alleles with the protective P9 conformation may interact more efficiently with the specific repertoire of T-cell receptors or T-cell subsets that respond to Bd antigens [52]. Many individuals with the advantageous P9 pocket conformation nonetheless died. The protective effect of MHC class II β1 molecules may be modulated by other factors regulating immune system function. The high virulence of the Bd strain to the frogs, indicated by massive infection loads borne by some subjects (figure 2; electronic supplementary material, table S6), may have resulted in effective inhibition of adaptive immunity [51].

Results from wild *L. v. alpina* populations show only weak evidence of selection for specific alleles of the MHC class II locus in infected populations. Inhibition of immune response may prevent selection of advantageous MHC alleles in the wild. However, the differential survival of subjects from the two historically infected sites suggests that selection for resistance to Bd varies among sites. Genetic variation and environmental factors may influence how strongly resistance alleles are selected in infected populations.

Overall disease resistance may correspond to levels of MHC heterozygosity [10,49]. The variation at the P9 pocket residues that we observed in amphibian populations long exposed to Bd is consistent with this view, although we did not observe any evidence of heterozygote advantage in our infection experiment. Amphibian populations with high frequencies of resistance alleles together with substantial MHC heterozygosity should be those most likely to recover from infection by Bd. Yet resistance alleles may have pleiotropic deleterious effects, including decreased growth, development or survival [8]. Heterozygosity may mitigate these effects in some conditions. It also may afford enhanced resistance against different Bd strains, as well as other fungal, bacterial and viral pathogens [8,9]. However, heterozygotes are not always more resistant than homozygotes to single infectious pathogens [53] such as Bd.

Further work is needed to assess the prevalence of the three pocket conformations in other Bd-resistant species. Also, our work suggests that the frequency of these conformations should increase in susceptible species as populations recover from chytridiomycosis epizootics. These MHC markers may make possible the identification of those amphibian populations most susceptible to Bd. Knowing which species are most at risk should facilitate the prioritization of conservation efforts and allow more accurate projections as to how Bd will affect complex host assemblages [54]. For those species now dependent on *ex situ* management for their very survival, selective breeding for Bd resistance may make possible successful re-introductions even into those areas in which Bd is enzootic. The molecular bases of resistance also may be critical to the development of immunization strategies. But much remains to be learned before these actions become practicable.

For most species included in our study, we have compared MHC-II β1 sequences obtained from one specific locus. Yet the MHC complex of many amphibians consists of at least two class II loci [14,15]. The structure and role of those additional loci in conferring disease resistance need to be further studied. In addition, the capacity of Bd to produce factors inhibiting amphibian adaptive immune response represents a major issue for any mitigation strategy based on adaptive immunity [51] that needs to be further elucidated.

Recent studies demonstrate that innate and adaptive immune defence systems can confer resistance against Bd [6,55,56]. Our study demonstrates for the first time that susceptibility to chytridiomycosis is associated with selection for specific immunogenetic traits across a wide variety of amphibians. Around the world, some amphibian species appear to have evolved immunity to chytridiomycosis through a common mechanism. Rescuing amphibian biodiversity will depend on our understanding of amphibian innate and adaptive immune defence mechanisms against Bd. The identification of adaptive markers for Bd resistance is an important step forward towards that goal.

## Supplementary Material

Supplementary materials

## Data accessibility

DNA sequences: GenBank accession numbers KJ679286–KJ679337. Microsatellite and MHC genotyping data for *Litoria verreauxii alpina* wild populations available from Dryad Digital Repository.
