# Supplementary material for: Susceptibility of amphibians to chytridiomycosis is associated with MHC class II conformation
Source: Proc Biol Sci. 2015 Apr 22;282(1805):20143127. doi: 10.1098/rspb.2014.3127 (PMC4389617; doi:10.1098/rspb.2014.3127)
Supplement: Supplementary materials [file rspb20143127supp1.pdf]

Supplementary methods

1) Isolation and characterization of MHC class II β1 domain

We collected fresh roadkills of *B. orientalis* and dissected out liver tissue which we kept at -80 °C for subsequent RNA extraction. RNA was extracted from liver samples using the RNeasy kit (Qiagen, Valencia, CA, USA) following the manufacturer's instructions. We also obtained liver samples from 10 *L. v. alpina* subjects that did not survive the experiment (see below) and were preserved in RNAlater (Life Technologies, Carlsbad, CA, USA) for subsequent RNA extraction.

RNA was used to synthesise complementary DNA (cDNA) by reverse transcription reaction using the ProtoScript AMV First Strand cDNA Synthesis Kit (New England BioLabs, Ipswich, MA, USA). The cDNA was used to amplify expressed MHC class II β1 alleles of *L. v. alpina* and *B. orientalis* by PCR using exonic primers BC6F and BobomSR (1), and BobomMHCIIEXON2F2 and BobomEXON2R1 (2), respectively. The resulting sequences were used to design primers to recover locus-specific intronic sequences flanking the MHC class II β1 domain using a genome walking technique (3) for *L. v. alpina* and an inverse PCR protocol (4) for *B. orientalis*.

The 5' intronic sequence of one MHCII β1 locus was obtained for *L. v. alpina* (GenBank accession number KJ679286) and the 3' intronic sequence was isolated for *B. orientalis* (GenBank accession number KJ679287). Using these sequences, we designed intronic primers (MHC-LVAintF1 and MHC-BOintR) that we used in combination with exonic reverse primer BobomSR (for *L. v. alpina*) and newly designed forward primer MHC-BOexF (for *B. orientalis*) to amplify by PCR locus-specific MHC class II β1 alleles for both species. Full details of primer sequences and PCR protocols are given in Table S2. Sequencing was done by the National Instrumentation Center for Environmental Management (NICEM, Seoul National University, South Korea).

2) Microsatellite markers isolation

In an attempt to identify the complete MHC gene complex of our target species and design primers with which we could genotype subjects, genomic DNA from targeted species was sheared and was hybridised with biotinylated probes to enriched it in MHC class I and class II sequences. The enriched genomic DNA was amplified and pyrosequenced using a 454-Roche instrument. Sequences obtained were screened to keep only good-quality sequences that were mined for MHC class I and class II in the program Geneious (5) using published amphibian MHC sequences as references. However, the method was unsuccessful in isolating MHC sequences (unpublished data). This partial genomic DNA library was mined for tandem repeat regions using MSATCOMMANDER (6). Primers were designed for 20 potential microsatellite markers, of which 7 gave single, scorable, polymorphic bands that were used in this study.

References

1. May S, Beebee T. 2009 Characterisation of major histocompatibility complex class II alleles in the natterjack toad, *Bufo calamita*. *Conserv. Genet. Resour.* **1**, 415-7. (doi: 10.1007/s12686-009-9096-6)
2. Hauswaldt J, Stuckas H, Pfautsch S, Tiedemann R. 2007 Molecular characterization of MHC class II in a nonmodel anuran species, the fire-bellied toad *Bombina bombina*. *Immunogenetics* **59**, 479-91. (doi: 10.1007/s00251-007-0210-1)
3. Cottage A, Yang A, Maunders H, de Lacy R, Ramsay N. 2001 Identification of DNA sequences flanking T-DNA insertions by PCR-walking. *Plant Mol. Biol. Rep.* **19**, 321-7. (doi: 10.1007/BF02772830)
4. Ochman H, Gerber AS, Hartl DL. 1988 Genetic applications of an inverse polymerase chain reaction. *Genetics* **120**, 621-3.
5. Geneious version 6.0 created by Biomatters. Available from http://www.geneious.com.
6. Faircloth BC. 2008 MSATCOMMANDER: detection of microsatellite repeat arrays and automated, locus-specific primer design. *Mol. Ecol. Resour.* **8**, 92-4. (doi: 10.1111/j.1471-8286.2007.01884)
7. May S, Zeisset I, Beebee T. 2011 Larval fitness and immunogenetic diversity in chytrid-infected and uninfected natterjack toad (*Bufo calamita*) populations. *Conserv Genet* 1-7.

Table S1. Genotyping results at β1 domain of MHC class II locus B of *Bufo gargarizans* and of one MHC class II locus of *Bombina orientalis.*

ID	Site	Sampling date	MHC-II β1 allele1	MHC-II β1 allele 2
Bufo gargarizans				
BG143	Jeonju	3/5/2012	2	3
BG144	Jeonju	3/5/2012	1	3
BG145	Jeonju	3/5/2012	3	6
BG168	Jeonju	3/8/2012	1	3
BG169	Jeonju	3/8/2012	2	3
BG172	Jeonju	3/9/2012	3	5
BG173	Jeonju	3/9/2012	1	3
BG194	Jeonju	3/11/2012	3	6
BG195	Jeonju	3/11/2012	1	5
BG196	Jeonju	3/11/2012	3	3
BG197	Jeonju	3/11/2012	6	6
BG198	Jeonju	3/11/2012	1	3
BG199	Jeonju	3/11/2012	2	6
BG200	Jeonju	3/11/2012	2	3
BG201	Jeonju	3/11/2012	1	3
BG202	Jeonju	3/11/2012	5	6
BG203	Jeonju	3/11/2012	3	6
BG204	Jeonju	3/11/2012	1	4
BG205	Jeonju	3/11/2012	1	2
BG206	Jeonju	3/11/2012	1	2
BG208	Jeonju	3/11/2012	3	6
BG209	Jeonju	3/11/2012	3	3
BG210	Jeonju	3/11/2012	2	3
BG211	Jeonju	3/11/2012	1	1
BG212	Jeonju	3/11/2012	3	5
BG213	Jeonju	3/11/2012	6	6
BG214	Jeonju	3/11/2012	7	7
BG215	Jeonju	3/11/2012	1	6
BG217	Jeonju	3/11/2012	2	3
BG218	Jeonju	3/11/2012	2	3
BG229	Geumsan	3/17/2012	1	5
BG230	Geumsan	3/17/2012	1	1
BG244	Geumsan	3/17/2012	4	5
BG245	Geumsan	3/17/2012	1	3
BG246	Geumsan	3/17/2012	5	5
BG247	Geumsan	3/17/2012	1	8
BG249	Geumsan	3/17/2012	1	3
BG251	Geumsan	3/20/2012	1	4
BG252	Geumsan	3/20/2012	1	3
BG253	Geumsan	3/20/2012	1	1
BG257	Geumsan	3/20/2012	3	5

Table S1. Continued

ID	Site	sampling date	MHC-II β1 allele1	MHC-II β1 allele 2
BG258	Geumsan	3/20/2012	1	2
BG259	Geumsan	3/20/2012	1	2
BG260	Geumsan	3/20/2012	1	5
BG261	Geumsan	3/20/2012	4	5
BG262	Geumsan	3/20/2012	3	3
BG263	Geumsan	3/20/2012	3	3
BG264	Geumsan	3/20/2012	3	4
BG272	Geumsan	3/20/2012	1	1
BG273	Geumsan	3/20/2012	1	1
BG275	Geumsan	3/20/2012	1	4
BG276	Geumsan	3/20/2012	1	1
BG277	Geumsan	3/20/2012	2	4
BG278	Geumsan	3/20/2012	1	1
BG279	Geumsan	3/20/2012	1	1
BG280	Geumsan	3/20/2012	3	4
BG281	Geumsan	3/20/2012	1	3
BG282	Geumsan	3/20/2012	1	3
BG284	Geumsan	3/20/2012	3	4
BG285	Geumsan	3/20/2012	3	3
Bombina orientalis				
BO060	Chuncheon	7/1/2011	1	2
BO061	Chuncheon	7/1/2011	6	2
BO064	Chuncheon	7/1/2011	3	1
BO065	Chuncheon	7/1/2011	2	2
BO066	Chuncheon	7/1/2011	3	1
BO067	Chuncheon	7/1/2011	7	1
BO068	Chuncheon	7/1/2011	3	3
BO069	Chuncheon	7/1/2011	1	1
BO070	Chuncheon	7/1/2011	4	1
BO071	Chuncheon	7/1/2011	1	1
BO272	Chiak	9/30/2011	3	3
BO273	Chiak	9/30/2011	3	3
BO274	Chiak	9/30/2011	1	2
BO275	Chiak	9/30/2011	3	3
BO276	Chiak	9/30/2011	2	1
BO277	Chiak	9/30/2011	3	1
BO278	Chiak	9/30/2011	3	1
BO279	Chiak	9/30/2011	5	2
BO280	Chiak	9/30/2011	3	1
BO281	Chiak	9/30/2011	3	3

Allele numbers correspond to *Buga*- and *Boor*- allele numbers in Fig. S1.

Table S2. List of primers and PCR protocols used for MHC isolation and genotyping.

Primer	sequence (5'-3')	Target	Reference	Conc (uM)	PCR cycling protocol
Litoria verreauxii alpina					
BCF6	CATTGTACAATCAGGAGGAG	MHC-II exon2	(1)	0.4	(94C for 40 sec, 54C for 40 sec, 72C for 1mi n)x35
bobomSR	CCATAGTTGTGTTTACAGACTGTTTCCAC	MHC-II exon2	(1)	0.4	
ADAPL	CTAATACGACTCACTATAGGGCTCGAGCGGCCGCCCGGGCAGGT	genome walking-adaptor	(3)		
ADPS	P-ACCTGCCC	genome walking-adaptor	(3)		
AP1	GGATCCTAATACGACTCACTATAGGGC	genome walking-1stPCR	(3)	0.2	(94C for 30 sec, 60C for 30 sec, 72C for 2 min)x35
LvaWP3	CTGTTCTATGATATCCTTATTGTTGT	genome walking-1stPCR-3'direction	this study	0.2	
LvaWP5	TATTTCATAGCGAAGACCGAGT	genome walking-1stPCR-5'direction	this study	0.2	
NAP1	TATAGGGCTCGAGCGGC	genome walking-2ndPCR	(3)	0.2	(94C for 30 sec, 60C for 30 sec, 72C for 2 min)x35*
LvaNWP3	CGCTGTCAAAGTAAAGAAACTC	genome walking-2ndPCR-3'direction	this study	0.2	
LvaNWP5	GAAACAGTCTGTAAACACAACTAT	genome walking-2ndPCR-5'direction	this study	0.2	
MHC-LVAintF1	GCTCTGGTCAGGGGAAATATT	Locus-specific MHC-II β1	this study	0.4	(94C for 30 sec, 58C for 30 sec, 72C for 1 min)x35
Bombina orientalis					
BobomMHCIIEXON2F2	CTGASTGTCACTTTATAAACGGCACTGA	MHC-II exon2	(2)	0.4	(94C for 40 sec, 50C for 40 sec, 72C for 1 min)x35
BobomMHCIIEXON2R1	CCATAGTTGTRTTTACAGACTGTTTCCAC	MHC-II exon2	(2)	0.4	
BOinvF	ACAGTRATAAGGACWWCATAGAG	inverse PCR	this study	0.2	(94C for 30 sec, 52C for 30 sec, 72C for 2 min)x35
BOinvR	ACCRACATCACTGTCAAAGT	inverse PCR	this study	0.2	
MHC-BOexF	TTTGACAGTGATGTYGGTAAAT	Locus-specific MHC-II β1	this study	0.2	(94C for 30 sec, 52C for 30 sec, 72C for 40 sec)x35
MHC-BOintR	GAAGGGTCATATAATGATATAGT	Locus-specific MHC-II β1	this study	0.2	
Bufo gargarizans					
2F347	GTGACCCTCTGCTCTCCATT	MHC-II locus β1	(7)	0.2	(94C for 30 sec, 58C for 30 sec, 72C for 40 sec)x35
2R307b	ATAATTCAGTATATACAGGGTCTCACC	MHC-II locus β1	(7)	0.2	
**M13**		Cloning		0.2	(94C for 30 sec, 52C for 30 sec, 72C for 1 min)x35

All PCR was performed with an Eppendorf 5331 Mastercycler PCR system in 20 μl volumes with 1 μl of DNA, 0.2 mM of each dNTP and 0.5 unit of TaKaRa Ex Taq polymerase, and started with a denaturation step at 94 C for 5 min and ended with an elongation step at 72C for 10min. (*) Second PCR starts with PCR product from first PCR diluted 1:100

Table S3. Characteristics of the 9 microsatellites markers used to genotype *Litoria verreauxii alpina* popμlations.

Locus name	Primer sequences (5'-3')	MP set	Ta	Repeat motif	Na	Allele size range (bp)	Freq NA	He	Ho	Accession number
LVmGT1	F: FAM-TAGACCGTATTTAACTAATGGGA	M1	52	(GT)9	14	166-192	0.150-0.342*	0.778-0.851	0.393-0.625	KJ679332
	R: ACGTTGAGCCTGATATCGC									
LVmAG1a	F: FAM-CTGCCCACTGCTCTGATAGTT	M1	52	(AG)9	6	212-226	(-)0.023-0.07	0.570-0.725	0.583-0.889	KJ679333
	R: GTGGGCGAAGACTAGCTGTT									
LVmAG1b	(same primers)	M1	52		9	227-247	0.059-0.098	0.695-0.812	0.600-0.692	KJ679333
LVmAG3	F: HEX-ACGTCACTTGTTGCTGGGAG	M1	52	(AG)8	9	236-266	0.039-0.700*	0.503-0.629	0.095-0.600	KJ679334
	R: TTCAAGCAAGAGCTTCGGCG									
LVmAG4	F: FAM-ATCCAGACTGACGCATGGCA	M2	55	(AG)6	7	357-373	(-)0.081-0.079	0.267-0.669	0.310-0.586	KJ679335
	R: AGAACCGTAAACCATTCAAGGC									
LVmAC4	F: HEX-CTGTAGTGACTGTAGGGTCATA	M2	55	(AC)10	7	218-232	0.047-0.068	0.620-0.693	0.591-0.621	KJ679336
	R: TGGTCTGTATCTTGGCCTGC									
LVmCCT1	F: FAM-CCGTGTGATTACTGGCTGCG	M2	55	(CCT)5	4	154-172	(-)0.03-0.014	0.100-0.625	0.103-0.615	KJ679337
	R:TGCCTGTCCTCTGACCATCC									
Le2*	F: HEX-TCTCCAGGACACAACACAGG	M1	52	(GT)11	10	189-208	0.063-0.273*	0.800-0.820	0.444-0.880	JF772865
	R: TTCCCTCTAGTGCCAACTGC									
Le4*	F: HEX-AATCCATCTCCGGGAATCTC	M2	55	(CA)11	6	155-169	0.062-0.269*	0.641-0.741	0.370-0.655	JF772868
	R: CTCGATCTTCGGTCTTGTGG									

MP set, multiplex set to each locus belongs; Ta, annealing temperature; Na, number of alleles; Freq NA, frequency of null alleles; He, expected heterozygosity; Ho, observed heterozygosity. Values encompass the three populations genotyped. (*) Loci with high frequency of null alleles (F > 0.10) also deviated from Hardy–Weinberg equilibrium. There was no linkage disequilibrium between loci.
Multiplex PCRs were performed in a 2-μl volume, containing approximately 10 ng of dried down DNA, 0.2–0.3 mM of each primer and 1 ml of QIAGEN Multiplex PCR Master Mix. The PCR program used was 95 C for 15 min, followed by 35 cycles of 94 C for 30 s, Ta for 90 s, 72 C for 60 s, and finally 60 C for 30 min; done on an Eppendorf 5331 Mastercycler PCR system. (*) Loci isolated and described in (45).

Table S4. Experimental design. Numbers of *L. v. alpina* frogs from each population and clutch, and details of allocation of treatment groups (exposed frogs versus sham-exposed negative control frogs).

Population (total number frogs)[a]	Clutch (total number frogs)[a]	Exposure group[b]	Total number of frogs	Number of males[f]	Number of females[f]	Number with undetermined gender
Greymare (80)	A (14)	E	14	7	6	1
		C	0	0	0	0
	B (26)	E	20	10	10	0
		C	6	3	3	0
	C (29)	E	20	11	9	0
		C	9	6	3	0
	D (11)	E	11	9	2	0
		C	0	0	0	0
Kiandra (100)	A (25)	E	20	9	11	0
		C	5	1	4	0
	B (25)	E	20	12	8	0
		C	5	1	4	0
	C (25)	E	20[c]	8	10	2
		C	5	3	2	0
	D (25)	E	20	11	9	0
		C	5	1	4	0
Ogilvies (76)	A (19)	E	19	13	6	0
		C	0	0	0	0
	B (40)	E	20	7	13	0
		C	20	6	14	0
	C (17)	E	16	9	7	0
		C	1[d]	0	0	1
Total	256		E (200)<br>C (56)[e]	127	125	4

[a]Total number of frogs in parentheses for each group; [b]E represents exposed frogs, C represents control frogs; [c]This number includes a frog that died of anasarca post-exposure, unrelated to chytridiomycosis; [d]This frog died pre-exposure and was excluded from analyses; [e]Total frogs grouped by exposure (E) and control (C); [f]Gender as determined by post-mortem coelomic examination. As our question of greatest interest involved comparison of infected frogs between populations, we maximised the sample size of exposed frogs by randomly selecting up to 20 frogs from each block (clutches within populations) to be exposed to Bd. Numbers of frogs available for treatments were subject to actual clutch sizes and natural attrition during growth and development. The control group was defined to ascertain the presence and nature of any cross-contamination in the experimental

setup. Some clutches where few frogs were available were of insufficient sample size to warrant the allocation of negative control individuals.

Table S5. Evidence of positive and purifying selection among potential peptide binding residues of amphibian MHC class II β1 domain

**Method**	**Pocket 4**	**Pocket 6**	**Pocket 4/7**	**Pocket 6/7**	**Pocket 9**
**Bufo**					
SLAC			67β*		_(-)37β**_
			71β*		_(-)56β*_
REL	13β**	11β**	67β**	30β**	57β**
	26β**	66β**	71β**		60β**
	74β**				_(-)37β*_
	78β**				_(-)56β**_
FEL	13β*	11β**	67β**	28β**	57β**
	26β*	66β*	71β*	30β*	_(-)37β*_
	74β*				_(-)56β**_
MEME	13β**	11β**	67β*	28β*	57β**
	74β**		71β*		60β**
**Bombina**					
SLAC	_(-)26β*_				
REL	_(-)26β*_		67β*		37β**
					57β*
					60β*
FEL	_(-)26β*_				37β*
MEME			67β*		37β**
			71β*		57β*
**Xenopus**					
REL			67β**		37β**
			71β*		
MEME			71β*		57β**
**Ambystoma**					
REL	13β**	11β**	67β**	28β**	37β**
	26β**		71β**	30β**	57β**
	74β**				
	78β**				
MEME	13β*		71β*		57β*
**Rana**					
SLAC					56β**
REL	26β**			28β**	37β**
				30β**	56β**
FEL	26β*			28β*	56β**
MEME	26β*			28β*	56β*
				30β*	
**Anda**					
REL	78β**	11β**	67β**	28β**	57β**
			71β**		
**Livea**					
REL	13β**	11β**	67β**	28β**	37β**
		66β**			57β**
FEL	13β*	11β*	67β*		
MEME	13β*	11β*	67β*	28β*	57β**

Bufo, orthologous MHC class II locus B alleles isolated from *Bufo bufo, B. calamita*, and *B. gargarizans; Bombina,* locus non-specific alleles from *Bombina bombina, B. variegata,* and *B. pachypus*; *Xenopus*, MHC-II β1 alleles isolated from *Xenopus laevis; Anda*, *Andrias davidianus*; *Ambystoma*, *A. mexicanum* and *A. tigrinum; Rana*, *R. yavapaiensis*, *R. catesbeiana*, *R. clamitans, R. pipiens, R. sylvatica, R. warszewitschii, R. temporaria;* *Livea*, *Litoria verreauxii alpina* individuals experimentally infected by *Bd*; MHC-II β1 alleles isolated from SLAC, single likelihood ancestor counting; REL, random-effects likelihood; FEL, fixed-effects likelihood; MEME, mixed-effects model of episodic diversifying selection; (*) 0.05> *P* > 0.01, posterior probability > 0.95 (for REL analysis); (**) *P* < 0.01, posterior probability > 0.99 (for REL analysis). All residues listed were identified as being under positive selection, except residues in italic and preceded by a negative sign.

Table S6. List of *Litoria verreauxii alpina* individuals from Bd infection experiment genotyped at the β1 domain of one MHC class II locus.

ID	Site	Clutch	Tank	Start Mass (g)	End Mass (g)	Mass Diff (g)	SVL (mm)	Max Inf (ZGE)	sex	Survived?	Survival Time (day)	P9+	P6+	Het	MHC-II β1 Allele1	MHC- II β1 Allele2
9	A	C	1	2.61	3.11	-0.5	30.8	112736	f	Yes	72	2/2	2/2	ho	5a	5a
12	A	C	1	2.79	2.04	0.75	29	350862	m	No	40	2/2	2/2	ho	5a	5a
30	A	D	2	7.58	5.56	2.02	38.2	787576	f	No	39	1/2	1/2	he	5b	8b
36	A	A	1	3.72	3.69	0.03	33.5	116102	f	No	31	0/2	0/2	ho	8a	8a
67	A	A	2	5.53	5.69	-0.16	36.1	76648	f	No	28	1/2	1/2	he	21	10
74	A	D	4	6.17	4.93	1.24	35.4	3285076	f	No	26	2/2	0/2	ho	14	14
100	A	C	4	2.3	2.62	-0.32	29	37471	m	No	31	2/2	2/2	ho	5a	5a
109	A	C	1	3.44	2.94	0.5	31.6	5612799	f	No	30	0/2	0/2	he	4	8a
118	A	B	2	4.18	3.54	0.64	34.3	36534	f	No	45	2/2	0/2	he	1	14
148	A	B	4	3.17	3.49	-0.32	32.3	57519	f	No	34	1/2	0/2	he	7a	13
170	A	A	1	6.1	5.75	0.35	34	2431749	f	No	36	1/2	0/2	he	8a	11
187	A	D	1	3.19	2.66	0.53	29.3	127486	m	No	39	1/2	1/2	he	5b	8b
199	A	B	2	2.16	2.11	0.05	26.5	322260	m	No	29	0/2	0/2	he	7a	8a
210	A	C	4	4.33	3.29	1.04	34.5	1665650	f	No	35	0/2	0/2	he	7a	8a
215	A	C	2	2.37	2.46	-0.09	28.7	577810	f	No	35	1/2	1/2	he	5a	8a
249	A	D	2	5.66	4.58	1.08	33.6	1264450	f	No	30	2/2	2/2	ho	5b	5b
260	A	B	3	3.26	2.75	0.51	29.8	18517	m	No	30	0/2	0/2	he	7a	8a
277	A	D	1	5.17	4.99	0.18	34.2	2463656	f	No	26	1/2	1/2	he	5b	8b
297	A	C	1	2.63	2.78	-0.15	27.8	184012	f	No	38	0/2	0/2	he	7a	8a
300	A	B	1	2.47	2.18	0.29	28.4	187494	f	No	38	1/2	1/2	he	5a	8a
330	A	B	1	3.81	3.74	0.07	30.9	663235	m	No	32	0/2	0/2	he	6	15
336	A	C	2	6.41	7.3	-0.89	33.5	664218	f	No	31	0/2	0/2	he	7a	8a
341	A	C	2	4.07	4.11	-0.04	30.7	447596	f	No	30	0/2	0/2	he	7a	8a
352	A	D	1	4.36	3.19	1.17	35	929209	f	No	33	2/2	2/2	ho	5b	5b
354	A	A	2	4.73	4.28	0.45	35.1	147473	f	No	48	0/2	0/2	he	6	7a
368	A	B	4	5.25	3.95	1.3	35.5	2149610	f	No	42	0/2	0/2	he	7a	8a
372	A	B	2	3.6	3.28	0.32	30.1	3228	f	Yes	72	2/2	0/2	he	1	13
383	A	C	4	2.76	2.32	0.44	28.4	712933	m	No	63	0/2	0/2	he	7a	8a
385	A	A	1	4.79	4.27	0.52	31.3	51733	m	No	23	1/2	0/2	he	2	7a
387	A	A	3	4.53	4.55	-0.02	33.9	529909	f	No	39	0/2	0/2	he	6	7a
394	A	A	3	4.59	3.91	0.68	32.8	8582	f	Yes	72	2/2	1/2	he	5b	11
397	A	D	1	2.41	3.25	-0.84	26.6	44137	f	Yes	72	2/2	0/2	he	2	14
400	A	A	3	3.67	3.49	0.18	29.9	853	m	Yes	72	2/2	1/2	he	3b	5a
2	B	C	1	2.2	2.25	-0.05	27.2	412545	m	No	36	0/2	0/2	ho	4	4
17	B	B	4	3.87	3.63	0.24	29.7	855307	m	No	22	0/2	0/2	he	8a	18
18	B	A	1	3.43	2.97	0.46	31.1	399523	m	No	28	1/2	0/2	he	3b	4
20	B	C	1	1.61	1.31	0.3	23.8	1163310	m	No	29	1/2	0/2	he	3a	4
45	B	B	3	4.5	4.23	0.27	33.4	828342	f	No	24	2/2	1/2	he	5c	12
47	B	B	1	3.46	3.8	-0.34	30.9	1241178	f	No	21	1/2	0/2	he	8a	12
72	B	C	1	4.49	4.22	0.27	34.6	492805	f	No	37	0/2	0/2	ho	4	4
89	B	A	1	2.1	1.81	-0.29	25.8	366371	m	No	23	1/2	1/2	he	4	5a
106	B	C	1	2.79	2.02	-0.77	28.1	387068	m	No	35	1/2	0/2	he	3a	4
120	B	A	3	2.51	2.18	-0.33	27.8	640914	m	No	29	0/2	0/2	ho	10	10
129	B	B	3	4.66	3.61	-1.05	33.1	830377	f	No	30	2/2	1/2	he	5b	12
136	B	A	1	2.17	1.33	-0.84	26.2	380071	m	No	32	1/2	0/2	he	3b	10
151	B	C	1	3.64	3.65	0.01	30.3	620529	f	No	28	2/2	0/2	ho	3a	3a
196	B	B	3	3.85	4.43	0.58	31.5	562288	f	No	23	2/2	2/2	ho	5b	5b
227	B	C	1	1.57	1.58	0.01	24.6	1413768	f	No	25	2/2	0/2	ho	3a	3a
248	B	B	4	5.42	4.72	-0.7	34.5	5970139	f	No	26	1/2	0/2	he	8a	13
258	B	A	3	3.81	3.28	-0.53	31.6	2291061	f	No	25	1/2	1/2	he	4	5a
261	B	B	2	4.91	3.77	-1.14	32	2512094	f	No	36	1/2	0/2	he	4	12
263	B	A	3	3	3.14	0.14	29.9	277552	m	No	23	1/2	1/2	he	4	5a
281	B	B	2	3.59	3.36	-0.23	29	2815366	m	No	26	2/2	1/2	he	5b	12
295	B	A	3	1.78	1.69	-0.09	27.2	1992837	m	No	23	1/2	0/2	he	3b	4
324	B	A	1	3.61	3.8	0.19	33.5	800314	f	No	24	1/2	0/2	he	3b	10
331	B	A	3	3.43	3.21	-0.22	30.7	829177	f	No	23	1/2	0/2	he	3b	10

Table S6. Continued

ID	Site	Clutch	Tank	Start Mass (g)	End Mass (g)	Mass Diff (g)	SVL (mm)	Max Inf (ZGE)	Sex	Survived?	Survival Time (day)	P9	P6	Het	MHC-II β1 Allele1	MHC-II β1 Allele2
333	B	C	1	3.54	3.13	-0.41	32.1	733436	f	No	34	0/2	1/2	ho	4	5a
335	B	B	1	2.78	2.5	-0.28	27.4	1387106	m	No	27	1/2	1/2	he	5b	4
339	B	A	1	4.01	3.27	-0.74	29.5	1947171	m	No	25	0/2	0/2	he	4	10
350	B	C	1	3.36	3.33	-0.03	29.8	552079	f	No	24	0/2	0/2	he	4	12
19	C	B	4	3.38	3.26	0.12	31	460153	f	No	25	1/2	1/2	he	5c	10
23	C	D	1	3.74	4	-0.26	29.4	1152590	m	No	25	1/2	0/2	he	1	3a
25	C	B	3	3.91	4.6	-0.69	31.6	45778	f	No	20	2/2	2/2	he	5c	5b
35	C	C	1	5.27	3.89	1.38	35.5	1289907	f	No	27	2/2	0/2	ho	1	3a
52	C	B	1	3.53	3.75	-0.22	32.2	538548	m	No	32	2/2	1/2	he	5c	13
53	C	C	3	3.84	3.65	0.19	33.2	2162675	m	No	28	2/2	1/2	he	1	5a
54	C	A	1	3.68	3.45	0.23	30.1	675129	m	No	26	2/2	0/2	he	1	12
96	C	A	1	5.81	5.78	0.03	33.9	67862	f	No	28	2/2	1/2	he	1	5a
111	C	C	2	3.49	2.88	0.61	30.9	489031	m	No	59	2/2	0/2	he	1	12
126	C	B	1	4.59	4.18	0.41	34.8	1838958	f	No	22	2/2	0/2	ho	13	4
131	C	A	1	3.28	2.82	0.46	28.1	6179549	m	No	23	2/2	1/2	ho	5c	10
154	C	B	4	4.84	5.2	-0.36	35.7	554706	f	No	31	2/2	0/2	ho	13	10
216	C	C	2	3.57	3.46	0.11	31	551506	m	No	60	1/2	0/2	he	3b	4
219	C	A	1	4.93	3.95	0.98	33.2	967513	f	No	24	2/2	1/2	ho	5c	6
222	C	D	1	7.05	6.24	0.81	37.8	1161688	f	No	24	1/2	1/2	he	5c	8a
225	C	C	3	3.64	3.41	0.23	32.7	652888	m	No	23	2/2	0/2	ho	3b	6
226	C	C	1	3.39	3.67	-0.28	31.2	584615	m	No	22	2/2	0/2	he	1	8b
230	C	A	1	4.83	3.4	1.43	32.6	316548	NA	No	29	1/2	0/2	he	3a	8a
239	C	C	2	3.58	3.83	-0.25	31.9	27	m	Yes	72	2/2	0/2	he	1	13
287	C	C	3	6.17	4.97	1.2	39.2	609344	f	No	33	2/2	0/2	ho	1	1
290	C	C	2	5.64	5.04	0.6	37.4	2531427	f	No	37	2/2	0/2	ho	1	13
344	C	B	3	4.29	4.41	-0.12	32.6	707072	f	No	32	2/2	0/2	he	13	3b
366	C	D	1	3.88	5.42	-1.54	30.6	329202	m	No	19	1/2	2/2	he	5b	5c
403	C	D	1	4.04	3.58	0.46	30.5	13660	m	No	32	2/2	1/2	he	11	5c

ID, frog individual number; Site, sampling site; Start Mass, mass at day=0 of experiment; End Mass, mass at death of day=72 of experiment; Mass Diff, difference in mass between day=0 and day=72; SVL, snout-vent length; Max Inf, maximum Bd infection load during the course of infection in zoospore genomic equivalent; Sex, m= male, f= female; P9+/P6+, presence of 1 (1/2) or 2 (2/2) alleles with the residue composition at the pocket 9/pocket 6 of the peptide-binding groove of MHC-II β1 associated with increased resistance to Bd; Het, heterozygosity, he= heterozygote, ho= homozygote.

Table S7. Results of the Cox proportional hazard model that best fit the data from the *Litoria verreauxii alpina* Bd infection experiment

Best model: Surv(days survived, died) ~ P9 + P6 + P9:MaxInf + MassDiff + Clutch + Site
(LRT = 61.88, df = 13, P < 0.0001)
Sample size N = 100 (see Table S6)

Significance of the explanatory variables predicting survival

**Variable**	**Loglik**	**Chisq**	**df**	***P***
Full	-253.81			
P9	-262.81	7.460	2	0.024
P6	-259.07	10.536	2	0.005
P9:MaxInf	-266.27	14.397	3	0.002
MassDiff	-262.06	5.972	1	0.015
Clutch	-266.01	13.879	3	0.003
Site	-272.15	26.148	2	<0.001

Effect of explanatory variables on survival

**Variable**	**coef**	**exp(coef)**	**se(coef)**	**z**	***P***
P9 (1/2)	-0.097	0.908	0.429	-0.225	0.822
P9 (2/2)	-1.335	0.263	0.392	-3.407	<0.001
P6 (1/2)	-0.259	0.772	0.300	-0.864	0.388
P6 (2/2)	1.650	5.205	0.537	3.070	0.002
Clutch B	-0.067	0.935	0.336	-0.201	0.841
Clutch C	-1.283	0.277	0.358	-3.580	<0.001
Clutch D	0.148	1.160	0.436	0.340	0.734
MassDiff	-0.643	0.526	0.249	-2.584	0.010
Site A	-1.794	0.166	0.395	-4.546	<0.001
Site B	0.063	1.064	0.367	0.170	0.865
P9(0/2):MaxInf	2.987e-08	1.000	1.779e-07	0.168	0.867
P9(1/2):MaxInf	4.150e-07	1.000	2.134e-07	1.945	0.052
P9(2/2):MaxInf	5.768e-07	1.000	1.350e-07	4.271	<0.001

P6/P9, individuals with two (2/2), one (1/2), or no (0/2) MHC-II β1 alleles with the specific residue compositions of pocket P6 and P9, respectively; MaxInf, maximum Bd infection load during the course of infection; Mass Diff, difference in mass between day=0 and day=72; Clutch, clutch of egg from which individual originated; Site, sampling site; LRT, Likelihood Ratio Test; df, degree of freedom; *P*, probability value.

Significance of each explanatory variable was evaluated by analyses of deviance between the full model and single term-deleted models.

The effect of each explanatory variables on survival of individuals can be estimated using the exponentiated coefficient, exp(coef). For categorical variables, exp(coef) can be interpreted as the instantaneous relative risk of an event at any time, for an individual with the risk factor present compared with an individual with the risk factor absent, given that both individuals are the same on all other covariates. P9(0/2), Clutch A and Site C are used as baselines for the estimation of risk. For continuous variables, exp(coef) is the instantaneous relative risk of an event, at any time, for an individual with an increase of 1 in the value of the covariate compared with another individual, given both individuals are the same on all other covariates. A value of exp(coef) below 1 suggests a reduction of hazard, while a value above 1 suggests an increase of hazard. coef, regression coefficient; exp(coef), exponentiated coefficient; se(coef), standard error of the coefficient; z, Wald statistic asymptotically standard normal under the hypothesis that the regression coefficient is zero.

Table S8. Detection of loci under positive selection in pairs of *L. v. alpina* populations.

	All sites			Site A-B			Site A-C			Site B-C		
Locus	Het	Fst	*PP*	Het	Fst	*PP*	Het	Fst	*PP*	Het	Fst	*PP*
LVmGT1	0.90	0.06	0.39	0.91	0.06	0.63	0.91	0.07	0.60	0.87	0.05	0.44
LVmAG1a	0.68	0.04	0.28	0.73	0.04	0.49	0.66	0.01	0.27	0.67	0.07	0.68
LVmAG1b	0.78	0.02	0.03	0.81	0.02	0.28	0.79	0.03	0.29	0.74	0.00	0.04
LVmAG3	**0.96**	**0.32**	**1.00**	**0.93**	**0.28**	**1.00**	**0.99**	**0.33**	**1.00**	**0.96**	**0.34**	**1.00**
Le2	0.91	0.09	0.71	0.88	0.05	0.51	0.90	0.08	0.71	0.95	0.14	0.96
LVmAC4	0.77	0.08	0.52	0.77	0.09	0.78	0.75	0.06	0.52	0.78	0.08	0.69
LVmAG4	0.49	0.09	0.66	0.55	0.15	0.85	0.57	0.07	0.61	0.34	0.02	0.43
LVmCCT1	**0.40**	**0.26**	**0.97**	0.13	-0.01	0.18	0.54	0.27	0.97	**0.53**	**0.31**	**0.99**
Le4	0.87	0.13	0.90	0.87	0.14	0.95	0.89	0.16	0.94	0.84	0.10	0.78
MHC-II β1	**0.96**	**0.15**	**0.99**	**0.94**	**0.16**	**0.99**	**0.98**	**0.19**	**1.00**	0.94	0.09	0.85

	All sites			Site A-B			Site A-C			Site B-C		
Locus	Het	Fst	*PP*	Het	Fst	*PP*	Het	Fst	*PP*	Het	Fst	*PP*
AG1a	0.68	0.04	0.36	0.73	0.04	0.54	0.66	0.01	0.31	0.67	0.07	0.73
AG1b	0.76	0.01	0.06	0.79	0.02	0.34	0.77	0.02	0.30	0.71	0.00	0.12
AC4	0.73	0.10	0.75	0.74	0.12	0.89	0.70	0.08	0.73	0.74	0.09	0.79
AG4	0.49	0.09	0.73	0.55	0.15	0.89	0.57	0.07	0.63	0.34	0.02	0.47
CCT1	**0.40**	**0.26**	**0.98**	0.13	-0.01	0.16	0.54	0.27	0.97	**0.53**	**0.31**	**0.99**
MHC-II β1	**0.95**	**0.16**	**1.00**	**0.94**	**0.17**	**0.99**	**0.98**	**0.20**	**1.00**	0.94	0.11	0.94

Genetic variation among populations (F_{ST}) at MHC and microsatellite markers was calculated and compared to simulated expected F_{ST} values using Fdist implemented in Lositan. In the first section, microsatellite markers corrected for null alleles are included. In the second section, only loci with frequency of null alleles < 0.1 were analyzed. Markers with F_{ST} values significantly higher than the simulated distribution (*PP* > 0.95) were considered to be under positive selection (rows in bold). Het; expected heterozygosity.

Figure S1. Alignment of the β1 domain of the MHC class II in amphibians illustrating peptide binding residues. Species represented include *Litoria verreauxii alpina* (Livea), *Bufo gargarizans* (Buga), *Bombina orientalis* (Boor), *Bombina bombina* (Bobo), *Bombina variegate* (Bova), *Bombina pachypus* (Bopa), *Bufo bufo* (Bubu), *Bufo* [*Epidalea*] *calamita* (Buca), *Rana* [*Lithobates*] *yavapaiensis* (Raya), *R. catesbeiana* (Raca), *R. clamitans* (Racl), *R. pipiens* (Rapi), *R. sylvatica* (Rasy), *R. warszewitschii* (Rawa), *Alytes obstetricans* (*Alob*), *Xenopus laevis* (*Xela*), *Andrias davidianus* (*Anda*), *Ambystoma mexicanum* and *tigrinum* (*Anme/Amti*). GenBank accession numbers are indicated for each sequence beside the name of the allele. *Livea* alleles in bold were associated with individuals surviving Bd infection. *Livea* alleles in italics were isolated from wild animals only. Peptide binding residues are classified according to their association with specific pockets of the binding groove: P4 (#), P4/P7 ($), P6 (^), P6/P7 (+), and P9 (*).

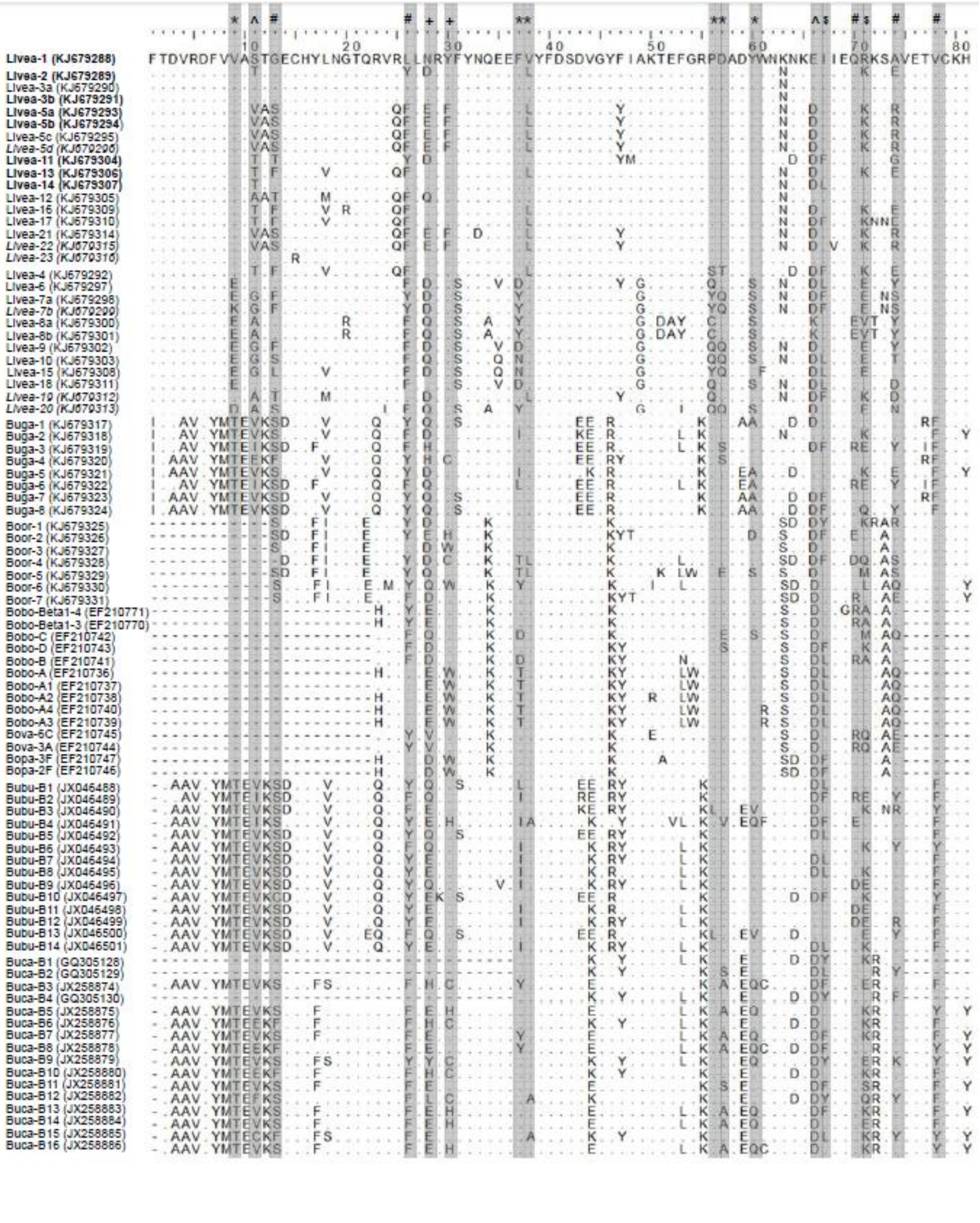

10 20 30 40 50 60 70 80
Livea-1 (KJ679288) FTDVRDFVVASTGECHYLNGTQRVRLLNRYFYNQEEFVYFDSDVGYF IAKTEFGRPDADYWNKNKEIIEQRKSAVETVCKH
Livea-2 (KJ679289)
Livea-3a (KJ679290)
Livea-3b (KJ679291)
Livea-5a (KJ679293)
Livea-5b (KJ679294)
Livea-5c (KJ679295)
Livea-5d (KJ679296)
Livea-11 (KJ679304)
Livea-13 (KJ679306)
Livea-14 (KJ679307)
Livea-12 (KJ679305)
Livea-16 (KJ679309)
Livea-17 (KJ679310)
Livea-21 (KJ679314)
Livea-22 (KJ679315)
Livea-23 (KJ679316)
Livea-4 (KJ679292)
Livea-6 (KJ679297)
Livea-7a (KJ679298)
Livea-7b (KJ679299)
Livea-8a (KJ679300)
Livea-8b (KJ679301)
Livea-9 (KJ679302)
Livea-10 (KJ679303)
Livea-15 (KJ679308)
Livea-18 (KJ679311)
Livea-19 (KJ679312)
Livea-20 (KJ679313)
Buga-1 (KJ679317)
Buga-2 (KJ679318)
Buga-3 (KJ679319)
Buga-4 (KJ679320)
Buga-5 (KJ679321)
Buga-6 (KJ679322)
Buga-7 (KJ679323)
Buga-8 (KJ679324)
Boor-1 (KJ679325)
Boor-2 (KJ679326)
Boor-3 (KJ679327)
Boor-4 (KJ679328)
Boor-5 (KJ679329)
Boor-6 (KJ679330)
Boor-7 (KJ679331)
Bobo-Beta1-4 (EF210771)
Bobo-Beta1-3 (EF210770)
Bobo-C (EF210742)
Bobo-D (EF210743)
Bobo-B (EF210741)
Bobo-A (EF210736)
Bobo-A1 (EF210737)
Bobo-A2 (EF210738)
Bobo-A4 (EF210740)
Bobo-A3 (EF210739)
Bova-6C (EF210745)
Bova-3A (EF210744)
Bopa-3F (EF210747)
Bopa-2F (EF210746)
Bubu-B1 (JX046488)
Bubu-B2 (JX046489)
Bubu-B3 (JX046490)
Bubu-B4 (JX046491)
Bubu-B5 (JX046492)
Bubu-B6 (JX046493)
Bubu-B7 (JX046494)
Bubu-B8 (JX046495)
Bubu-B9 (JX046496)
Bubu-B10 (JX046497)
Bubu-B11 (JX046498)
Bubu-B12 (JX046499)
Bubu-B13 (JX046500)
Bubu-B14 (JX046501)
Buca-B1 (GQ305128)
Buca-B2 (GQ305129)
Buca-B3 (JX258874)
Buca-B4 (GQ305130)
Buca-B5 (JX258875)
Buca-B6 (JX258876)
Buca-B7 (JX258877)
Buca-B8 (JX258878)
Buca-B9 (JX258879)
Buca-B10 (JX258880)
Buca-B11 (JX258881)
Buca-B12 (JX258882)
Buca-B13 (JX258883)
Buca-B14 (JX258884)
Buca-B15 (JX258885)
Buca-B16 (JX258886)

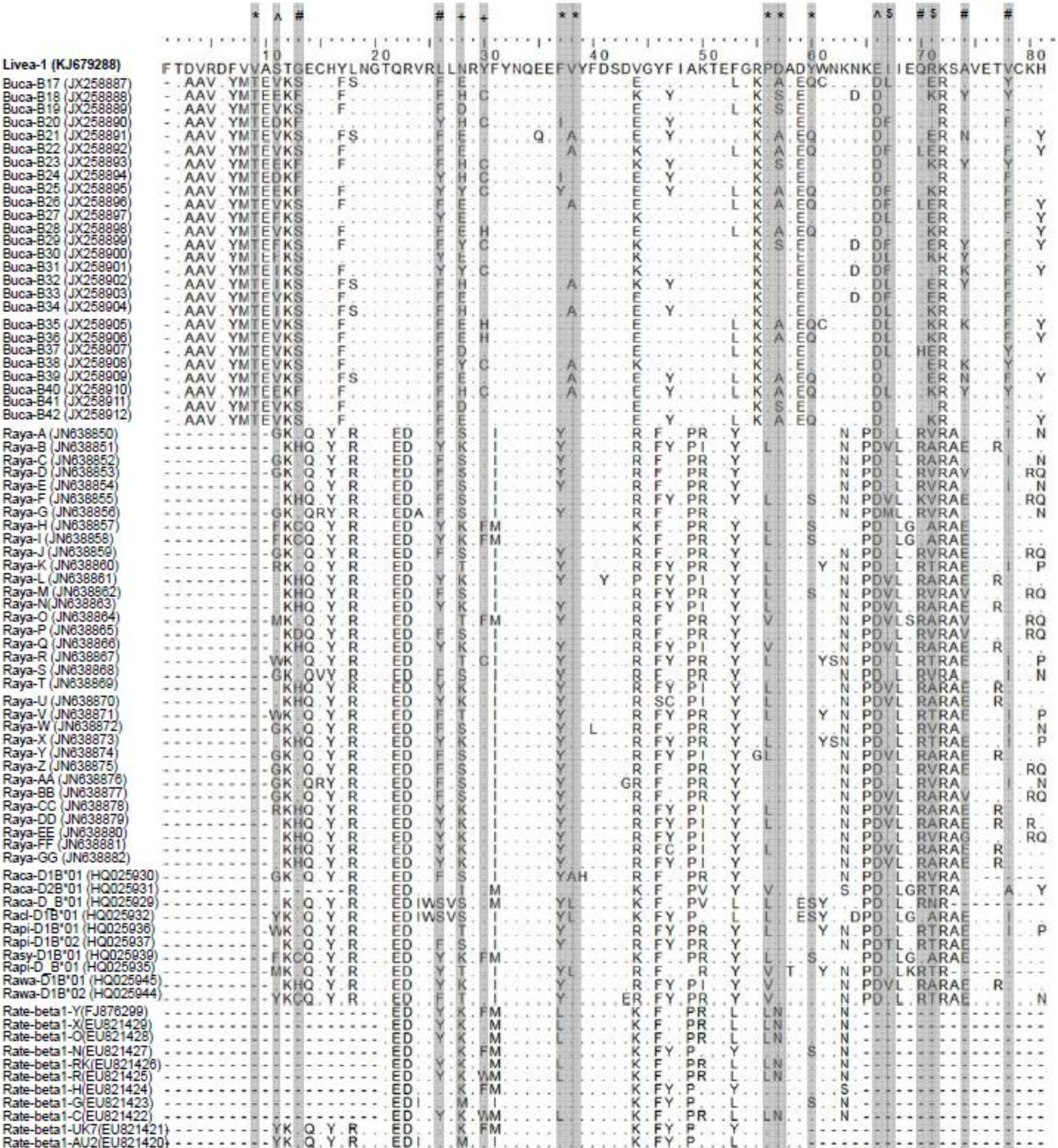
Livea-1 (KJ679288)
FTDVRDFVVASTGECHYLNGTQRVRLLNRYFYNQEEFVYFDSDVGYF IAKTEFGRPDADYWNKNKEIIEQRKSAVETVCKH
Buca-B17 (JX258887)
Buca-B18 (JX258888)
Buca-B19 (JX258889)
Buca-B20 (JX258890)
Buca-B21 (JX258891)
Buca-B22 (JX258892)
Buca-B23 (JX258893)
Buca-B24 (JX258894)
Buca-B25 (JX258895)
Buca-B26 (JX258896)
Buca-B27 (JX258897)
Buca-B28 (JX258898)
Buca-B29 (JX258899)
Buca-B30 (JX258900)
Buca-B31 (JX258901)
Buca-B32 (JX258902)
Buca-B33 (JX258903)
Buca-B34 (JX258904)
Buca-B35 (JX258905)
Buca-B36 (JX258906)
Buca-B37 (JX258907)
Buca-B38 (JX258908)
Buca-B39 (JX258909)
Buca-B40 (JX258910)
Buca-B41 (JX258911)
Buca-B42 (JX258912)
Raya-A (JN638850)
Raya-B (JN638851)
Raya-C (JN638852)
Raya-D (JN638853)
Raya-E (JN638854)
Raya-F (JN638855)
Raya-G (JN638856)
Raya-H (JN638857)
Raya-I (JN638858)
Raya-J (JN638859)
Raya-K (JN638860)
Raya-L (JN638861)
Raya-M (JN638862)
Raya-N(JN638863)
Raya-O (JN638864)
Raya-P (JN638865)
Raya-Q (JN638866)
Raya-R (JN638867)
Raya-S (JN638868)
Raya-T (JN638869)
Raya-U (JN638870)
Raya-V (JN638871)
Raya-W (JN638872)
Raya-X (JN638873)
Raya-Y (JN638874)
Raya-Z (JN638875)
Raya-AA (JN638876)
Raya-BB (JN638877)
Raya-CC (JN638878)
Raya-DD (JN638879)
Raya-EE (JN638880)
Raya-FF (JN638881)
Raya-GG (JN638882)
Raca-D1B*01 (HQ025930)
Raca-D2B*01 (HQ025931)
Raca-D_B*01 (HQ025929)
Racl-D1B*01 (HQ025932)
Rapi-D1B*01 (HQ025936)
Rapi-D1B*02 (HQ025937)
Rasy-D1B*01 (HQ025939)
Rapi-D_B*01 (HQ025935)
Rawa-D1B*01 (HQ025945)
Rawa-D1B*02 (HQ025944)
Rate-beta1-Y(FJ876299)
Rate-beta1-X(EU821429)
Rate-beta1-O(EU821428)
Rate-beta1-N(EU821427)
Rate-beta1-RK(EU821426)
Rate-beta1-R(EU821425)
Rate-beta1-H(EU821424)
Rate-beta1-G(EU821423)
Rate-beta1-C(EU821422)
Rate-beta1-UK7(EU821421)
Rate-beta1-AU2(EU821420)
Rate-3A(EF210761)
Rate-02-01(JN412623)
Rate-01-01(JN412622)

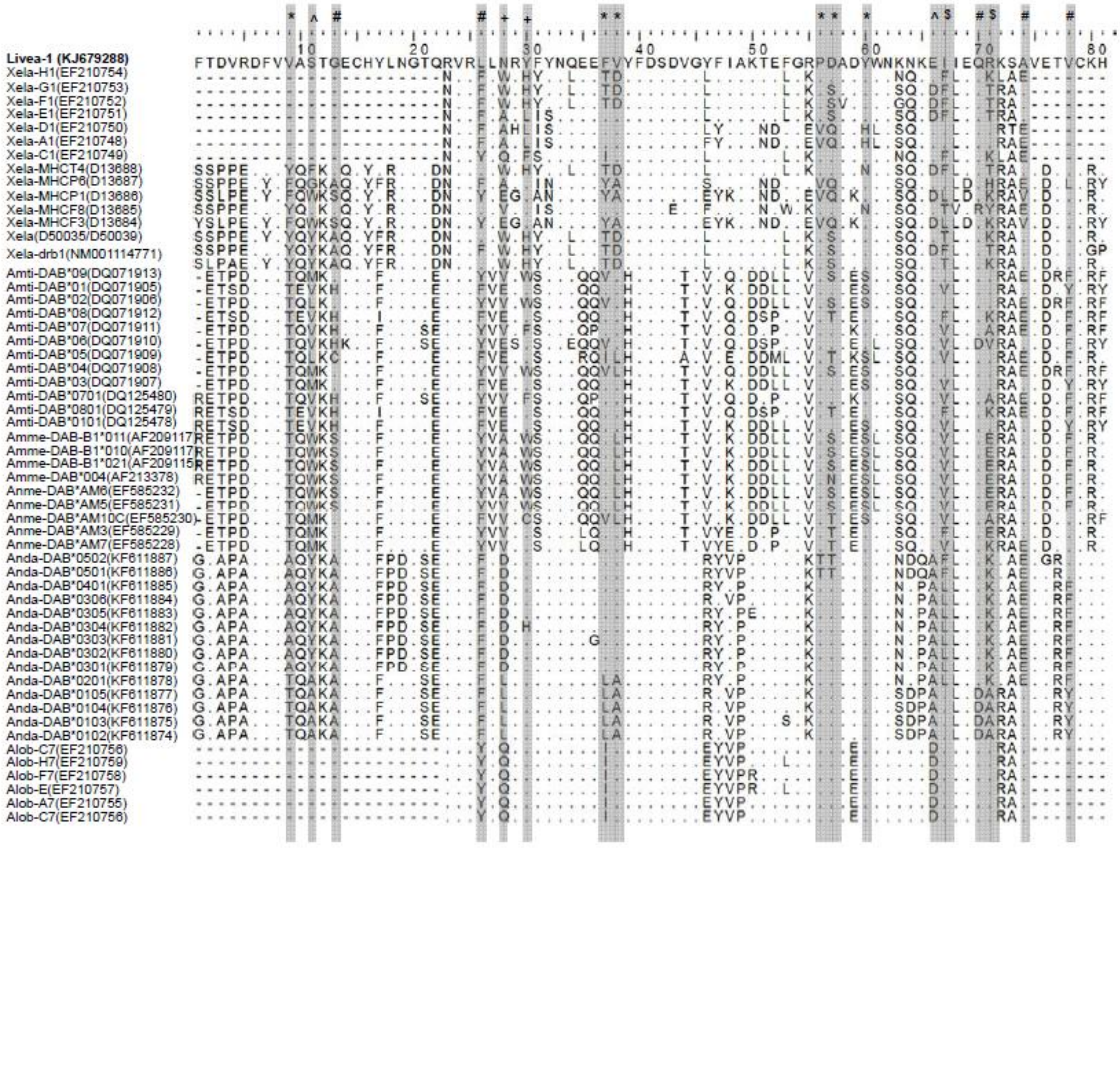
Livea-1 (KJ679288)
FTDVRDFVVASTGECHYLNGTQRVRLLNRYFYNQEEFVYFDSDVGYFIAKTEFGRPDADYWNKNKEIIEQRKSAVETVCKH
Xela-H1(EF210754)
Xela-G1(EF210753)
Xela-F1(EF210752)
Xela-E1(EF210751)
Xela-D1(EF210750)
Xela-A1(EF210748)
Xela-C1(EF210749)
Xela-MHCT4(D13688)
Xela-MHCP6(D13687)
Xela-MHCP1(D13686)
Xela-MHCF8(D13685)
Xela-MHCF3(D13684)
Xela(D50035/D50039)
Xela-drb1(NM001114771)
Amti-DAB*09(DQ071913)
Amti-DAB*01(DQ071905)
Amti-DAB*02(DQ071906)
Amti-DAB*08(DQ071912)
Amti-DAB*07(DQ071911)
Amti-DAB*06(DQ071910)
Amti-DAB*05(DQ071909)
Amti-DAB*04(DQ071908)
Amti-DAB*03(DQ071907)
Amti-DAB*0701(DQ125480)
Amti-DAB*0801(DQ125479)
Amti-DAB*0101(DQ125478)
Amme-DAB-B1*011(AF209117)
Amme-DAB-B1*010(AF209117)
Amme-DAB-B1*021(AF209115)
Amme-DAB*004(AF213378)
Anme-DAB*AM6(EF585232)
Anme-DAB*AM5(EF585231)
Anme-DAB*AM10C(EF585230)
Anme-DAB*AM3(EF585229)
Anme-DAB*AM7(EF585228)
Anda-DAB*0502(KF611887)
Anda-DAB*0501(KF611886)
Anda-DAB*0401(KF611885)
Anda-DAB*0306(KF611884)
Anda-DAB*0305(KF611883)
Anda-DAB*0304(KF611882)
Anda-DAB*0303(KF611881)
Anda-DAB*0302(KF611880)
Anda-DAB*0301(KF611879)
Anda-DAB*0201(KF611878)
Anda-DAB*0105(KF611877)
Anda-DAB*0104(KF611876)
Anda-DAB*0103(KF611875)
Anda-DAB*0102(KF611874)
Alob-C7(EF210756)
Alob-H7(EF210759)
Alob-F7(EF210758)
Alob-E(EF210757)
Alob-A7(EF210755)
Alob-C7(EF210756)

Database (will be submitted to DRYAD upon manuscript acceptance)

[Genotyping results for 9 microsatellite markers and the exon2 of one MHC class II locus for *Litoria verreauxii alpina* wild populations]

GT1
AG1a
AG1b
AG3
Le2
AC4
AG4
CCT1
Le4
MHC

POP

1 ,	178178	216220	235237	236236	191203	224224	359359
	158158	161161	000000				
2 ,	166178	220220	235237	236246	189206	232232	359359
	158160	159159	101205				
4 ,	166174	216220	227235	236236	197206	224228	359359
	158160	161161	101113				
6 ,	178178	000000	233237	236238	197206	224226	357359
	160160	159159	101101				
7 ,	174178	216216	237239	236238	197206	224232	357359
	160160	159161	103103				
9 ,	000000	216222	000000	236236	191197	228228	359359
	158160	159159	103122				
10 ,	176176	220220	233237	236246	197206	232232	359363
	158172	159159	305305				
11 ,	166176	216220	227235	238246	189197	224232	359363
	160172	159161	101305				
12 ,	166176	220220	235237	236246	199203	000000	359359
	158160	161161	101405				
13 ,	000000	000000	000000	000000	000000	000000	359359
	158172	159159	101305				
14 ,	178178	216220	235237	236236	189203	224224	359363
	160172	161163	101103				
16 ,	174180	216220	235239	236236	189197	224224	359363
	158160	161163	305112				
17 ,	174174	216222	235235	236236	197206	000000	359359
	160160	161163	103103				
18 ,	178178	216216	237237	238246	189189	232232	359359
	158158	161161	305305				
19 ,	178178	220220	235235	236238	189203	224232	359359
	160160	159159	125405				

20 ,	180180	216220	239239	238250	193197	226226	357359
	160160	161163	203203				
21 ,	166178	216216	237237	238246	189206	224224	359359
	158172	159159	101305				
22 ,	176176	216216	235235	236236	191197	228232	359359
	158172	161161	000000				
23 ,	166166	216220	233235	236246	206206	224224	357359
	160160	165167	305208				
24 ,	166166	220220	235235	246246	197206	218224	359359
	158160	161163	305305				
25 ,	166166	216220	235235	236236	206206	224224	359359
	160160	161163	103113				
26 ,	178178	216220	235237	236236	203206	224232	359359
	158172	161167	000000				
27 ,	166174	220220	227237	236246	197206	232232	359359
	160160	163167	103103				
28 ,	168182	216224	000000	246250	191193	224224	363365
	160172	161161	000000				
29 ,	166176	216220	235235	238246	197206	224224	359363
	158172	159163	000000				
Greymare ,	166178	216220	233237	236238	197203	226232	357359
	158160	163163	000000				
POP							
31 ,	000000	216220	235235	246246	189189	224224	357361
	160160	159169	000000				
32 ,	166182	216222	000000	258258	189197	000000	000000
	000000	000000	103109				
33 ,	166178	216222	233237	000000	189189	224228	359363
	160160	157157	111119				
34 ,	166190	216216	235239	246246	193206	224224	359359
	160160	000000	000000				
35 ,	184186	220226	233235	000000	203203	218224	359361
	160160	161161	205120				
36 ,	184184	216220	235235	258258	189193	228230	359359
	160160	157157	109109				
37 ,	180186	212220	235239	258264	195208	224230	359359
	154160	157157	205109				
38 ,	180186	216216	233239	000000	203203	224224	359363
	160160	157163	000000				
39 ,	178184	216220	237243	000000	189195	224224	357361
	160160	159159	109109				
40 ,	166186	224224	233247	258258	189199	222232	359359
	160160	157161	107207				
41 ,	178186	000000	237237	258258	203203	224230	361373
	160160	157159	109109				
42 ,	182182	220226	241243	258258	189197	224230	363363
	160160	157157	122109				

43 ,	166182	216220	235241	264264	189197	224224	359359
	160160	157161	122109				
44 ,	168178	220224	000000	264264	203203	224232	359359
	160160	157165	109109				
45 ,	178186	216216	000000	258258	195195	224224	361363
	154160	157159	109109				
46 ,	188190	216220	235235	000000	203203	230230	359371
	160160	159159	121109				
47 ,	186186	216224	233233	264264	189193	228230	359359
	160160	157157	000000				
48 ,	178184	216216	235243	000000	189206	224224	359363
	160160	163163	000000				
49 ,	184184	220224	239243	258258	193201	232232	357359
	160160	159163	205205				
50 ,	000000	216226	235235	264264	189189	224224	357359
	160160	157157	000000				
51 ,	184184	216222	233243	258258	195199	224230	359359
	160160	157157	000000				
52 ,	000000	216220	000000	000000	189195	224224	361371
	160160	157159	000000				
53 ,	166166	220226	235246	264264	197197	224230	361363
	160160	159159	104109				
54 ,	000000	216220	239241	000000	195199	224228	363363
	160160	000000	000000				
55 ,	000000	220220	235235	258258	189189	224230	359363
	160160	157157	205109				
56 ,	180188	220220	235239	000000	193195	230230	359363
	160172	159163	101107				
57 ,	000000	216220	237237	264264	203206	230230	359359
	154160	157157	000000				
58 ,	184184	220224	237243	258258	189189	224224	359363
	154160	157157	103109				
59 ,	186186	216222	235246	258264	189199	224224	361363
	160160	157157	122109				
Kiandra ,	186186	220224	235246	258258	195195	230230	359359
	160160	159159	122109				
POP							
61 ,	170182	220226	233233	000000	193203	224232	357359
	154160	157159	205205				
62 ,	182182	000000	237237	000000	201201	224232	357359
	154160	157159	113405				
63 ,	170170	216220	235235	000000	191191	224228	359359
	160160	157169	103103				
64 ,	178182	220226	233237	000000	189189	224232	359359
	160160	161161	000000				
65 ,	176178	216220	235237	246246	191191	224228	357359
	160160	155169	205205				

66 ,	170170	216220	235246	000000	193193	224228	359359
	160160	155161	000000				
67 ,	174178	216220	235237	000000	201201	228228	359359
	160160	161161	203203				
68 ,	180192	216220	235239	000000	189208	226226	359359
	160160	155157	103103				
69 ,	178184	216216	235237	250266	193208	224232	359359
	160160	155161	122112				
70 ,	182186	220226	241246	250250	189193	226228	359359
	160160	155161	205112				
71 ,	178182	220226	233237	000000	193208	224228	359359
	160160	169169	000000				
72 ,	178182	216220	233239	000000	189191	224228	359359
	160160	161161	103122				
73 ,	178178	220226	235237	000000	193193	228230	357359
	160160	155157	104205				
74 ,	176178	220226	233237	250264	189193	228228	359359
	160160	157161	110205				
75 ,	178178	220226	237237	264264	189193	228228	359359
	160160	157157	208112				
76 ,	170170	000000	235235	250250	201201	224232	359359
	160160	161161	000000				
77 ,	182182	216220	237237	250250	193203	224224	359359
	160160	155161	205205				
78 ,	178178	216220	235239	236240	208208	224230	357359
	160160	157161	101101				
79 ,	000000	220226	235235	250250	193193	228228	359359
	160160	161169	104205				
80 ,	174174	220226	235239	250250	000000	228230	359359
	160160	157169	000000				
81 ,	190192	220226	237237	250250	201201	224226	359359
	160160	161161	000000				
82 ,	186186	000000	237246	250266	189189	224224	357359
	160160	161161	103205				
83 ,	170170	216220	235235	250250	193193	224228	359359
	160160	167169	205108				
84 ,	178178	216226	235237	250250	193193	228228	357359
	160160	155161	205113				
85 ,	182182	220226	235237	250260	189189	228228	357359
	160160	157169	205113				
86 ,	186186	220226	237246	250250	000000	224228	359359
	160160	161161	000000				
87 ,	000000	220226	235235	250250	189203	224224	359359
	160160	155155	000000				
88 ,	178178	220220	235239	250250	193203	226228	359359
	160160	157161	205122				
89 ,	178178	224226	237237	250250	000000	000000	000000
	000000	000000	000000				

Ogilvies ,	192192	216216	233233	266266	201208	224224	357359	154160	157161	205108

[Numbers corresponds to allele size for microsatellite markers (see Table S5 for more information).]
[Codes used for MHC alleles: 101/Livea-1, 103/Livea-3a, 203/Livea-3b, 104/Livea-4, 205/Livea-5b, 305/Livea-5c, 405/Livea-5d, 107/Livea-7a, 207/Livea-7b, 108/Livea-8a, 208/Livea-8b, 109/Livea-9, 110/Livea-10, 112/Livea-12, 113/Livea-13, 119/Livea-19, 120/Livea-20, 121/Livea-21, 122/Livea-22]

[Genotyping results for 9 microsatellite markers and the exon2 of one MHC class II locus for *Litoria verreauxii alpina* wild populations-corrected for null alleles using the so-called INA method described in Chapuis and Estoup (2007), GENEPOP format]
loc1
loc2
loc3
loc4
loc5
loc6
loc7
loc8
loc9
loc10
POP
1, 0199 0102 0102 0101 0102 0199 0101 0101 0101 0000
1, 0201 0202 0102 0102 0304 0299 0101 0102 0202 0102
1, 0203 0102 0301 0101 0504 0103 0101 0102 0199 0103
1, 0199 0000 0402 0103 0504 0104 0201 0202 0202 0199
1, 0301 0101 0205 0103 0504 0102 0201 0202 0201 0499
1, 0000 0103 0000 0101 0105 0399 0101 0102 0299 0405
1, 0499 0202 0402 0102 0504 0202 0103 0103 0299 0606
1, 0204 0102 0301 0302 0305 0102 0103 0203 0201 0106
1, 0204 0202 0102 0102 0602 0000 0101 0102 0101 0107
1, 0000 0000 0000 0000 0000 0000 0101 0103 0202 0106
1, 0199 0102 0102 0101 0302 0101 0103 0203 0103 0104
1, 0305 0102 0105 0101 0305 0199 0103 0102 0103 0608
1, 0399 0103 0101 0101 0504 0000 0101 0202 0103 0404
1, 0101 0101 0202 0302 0303 0202 0101 0101 0199 0699
1, 0101 0202 0101 0103 0302 0102 0101 0202 0202 0907
1, 0599 0102 0599 0304 0705 0499 0201 0202 0103 1099
1, 0201 0101 0202 0302 0304 0199 0101 0103 0299 0106
1, 0499 0101 0101 0101 0105 0302 0101 0103 0101 0000
1, 0299 0102 0401 0102 0404 0101 0201 0202 0405 0611
1, 0299 0202 0101 0202 0504 0501 0101 0102 0103 0606
1, 0202 0102 0199 0101 0404 0101 0101 0202 0103 0403
1, 0101 0102 0102 0101 0204 0102 0101 0103 0105 0000
1, 0203 0202 0302 0102 0504 0299 0101 0202 0305 0499
1, 0607 0104 0000 0204 0107 0101 0304 0203 0101 0000
1, 0204 0102 0101 0302 0504 0101 0103 0103 0203 0000
Greymare, 0201 0102 0402 0103 0502 0402 0201 0102 0399 0000
POP
2, 0000 0102 0199 0299 0303 0101 0205 0202 0206 0000
2, 0207 0103 0000 0599 0305 0000 0000 0000 0000 0412
2, 0201 0103 0402 0000 0303 0103 0103 0202 0707 1314
2, 0208 0101 0105 0299 0704 0199 0101 0202 0000 0000
2, 0910 0205 0401 0000 0202 0501 0105 0202 0199 0215
2, 0909 0102 0101 0505 0307 0306 0101 0202 0707 1212
2, 0510 0602 0105 0506 0809 0106 0101 0402 0799 0212

2, 0510 0101 0405 0000 0299 0101 0103 0202 0703 0000
2, 0109 0102 0206 0000 0308 0199 0205 0202 0299 1212
2, 0210 0404 0407 0599 0306 0702 0101 0202 0701 1617
2, 0110 0000 0202 0599 0202 0106 0506 0202 0702 1212
2, 0799 0205 0806 0505 0305 0106 0303 0202 0707 0512
2, 0207 0102 0108 0699 0305 0199 0101 0202 0701 0512
2, 0601 0204 0000 0699 0299 0102 0101 0202 0704 1212
2, 0110 0101 0000 0599 0899 0101 0503 0402 0702 1212
2, 1108 0102 0101 0000 0299 0699 0107 0202 0299 1812
2, 1099 0104 0404 0699 0307 0306 0101 0202 0799 0000
2, 0109 0101 0106 0000 0304 0101 0103 0202 0399 0000
2, 0909 0204 0506 0505 0710 0299 0201 0202 0203 0202
2, 0000 0105 0101 0606 0399 0101 0201 0202 0799 0000
2, 0999 0103 0406 0599 0806 0106 0101 0202 0707 0000
2, 0000 0102 0000 0000 0308 0101 0507 0202 0702 0000
2, 0299 0205 0109 0699 0599 0106 0503 0202 0202 1912
2, 0000 0102 0508 0000 0806 0103 0303 0202 0000 0000
2, 0000 0202 0101 0599 0399 0106 0103 0202 0799 0212
2, 0511 0202 0105 0000 0708 0606 0103 0203 0203 0116
2, 0000 0102 0299 0606 0204 0606 0101 0402 0799 0000
2, 0999 0204 0206 0505 0303 0101 0103 0402 0707 0412
2, 1010 0103 0109 0506 0306 0101 0503 0202 0707 0512
Kiandra, 1010 0204 0109 0599 0808 0699 0101 0202 0202 0512
POP
3, 1207 0205 0499 0000 0702 0102 0201 0402 0702 0202
3, 0707 0000 0202 0000 1099 0102 0201 0402 0702 0307
3, 1299 0102 0199 0000 0199 0103 0101 0202 0706 0404
3, 0107 0205 0402 0000 0399 0102 0101 0202 0101 0000
3, 0401 0102 0102 0299 0199 0103 0201 0202 0806 0299
3, 1299 0102 0109 0000 0707 0103 0101 0202 0801 0000
3, 0301 0102 0102 0000 1099 0303 0101 0202 0101 1099
3, 0513 0102 0105 0000 0309 0404 0101 0202 0807 0499
3, 0109 0101 0102 0407 0709 0102 0101 0202 0801 0508
3, 0710 0205 0809 0404 0307 0403 0101 0202 0801 0208
3, 0107 0205 0402 0000 0709 0103 0101 0202 0606 0000
3, 0107 0102 0405 0000 0301 0103 0101 0202 0101 0405
3, 0199 0205 0102 0000 0799 0306 0201 0202 0807 1902
3, 0401 0205 0402 0406 0307 0303 0101 0202 0701 2002
3, 0199 0205 0299 0699 0307 0303 0101 0202 0707 1108
3, 1212 0000 0101 0499 1099 0102 0101 0202 0101 0000
3, 0799 0102 0202 0404 0702 0101 0101 0202 0801 0202
3, 0199 0102 0105 0108 0999 0106 0201 0202 0701 0199
3, 0000 0205 0101 0404 0799 0399 0101 0202 0106 1902
3, 0399 0205 0105 0404 0000 0306 0101 0202 0706 0000
3, 0813 0205 0202 0499 1010 0104 0101 0202 0101 0000
3, 1099 0000 0209 0407 0303 0199 0201 0202 0101 0402
3, 1299 0102 0101 0499 0707 0103 0101 0202 0506 0221
3, 0101 0105 0102 0499 0799 0303 0201 0202 0801 0203

3,	0799	0205	0102	0409	0399	0303	0201	0202	0706	0203
3,	1099	0205	0209	0499	0000	0103	0101	0202	0199	0000
3,	0000	0205	0101	0404	0302	0101	0101	0202	0808	0000
3,	0101	0202	0105	0404	0702	0403	0101	0202	0701	0205
3,	0199	0405	0202	0404	0000	0000	0000	0000	0000	0000
Ogilvies,	1399	0101	0404	0799	1009	0101	0201	0402	0701	0221
